# Stochastic Regulation of her1/7 Gene Expression Is the Source of Noise in the Zebrafish Somite Clock Counteracted by Notch Signalling

**DOI:** 10.1371/journal.pcbi.1004459

**Published:** 2015-11-20

**Authors:** Robert P. Jenkins, Anja Hanisch, Cristian Soza-Ried, Erik Sahai, Julian Lewis

**Affiliations:** 1 Tumour Cell Biology Laboratory, The Francis Crick Institute Lincoln’s Inn Fields Laboratory, London, United Kingdom; 2 Vertebrate Development Laboratory, The Francis Crick Institute Lincoln’s Inn Fields Laboratory, London, United Kingdom; University of California Irvine, UNITED STATES

## Abstract

The somite segmentation clock is a robust oscillator used to generate regularly-sized segments during early vertebrate embryogenesis. It has been proposed that the clocks of neighbouring cells are synchronised via inter-cellular Notch signalling, in order to overcome the effects of noisy gene expression. When Notch-dependent communication between cells fails, the clocks of individual cells operate erratically and lose synchrony over a period of about 5 to 8 segmentation clock cycles (2–3 hours in the zebrafish). Here, we quantitatively investigate the effects of stochasticity on cell synchrony, using mathematical modelling, to investigate the likely source of such noise. We find that variations in the transcription, translation and degradation rate of key Notch signalling regulators do not explain the *in vivo* kinetics of desynchronisation. Rather, the analysis predicts that clock desynchronisation, in the absence of Notch signalling, is due to the stochastic dissociation of Her1/7 repressor proteins from the oscillating *her1/7* autorepressed target genes. Using in situ hybridisation to visualise sites of active *her1* transcription, we measure an average delay of approximately three minutes between the times of activation of the two *her1* alleles in a cell. Our model shows that such a delay is sufficient to explain the *in vivo* rate of clock desynchronisation in Notch pathway mutant embryos and also that Notch-mediated synchronisation is sufficient to overcome this stochastic variation. This suggests that the stochastic nature of repressor/DNA dissociation is the major source of noise in the segmentation clock.

## Author Summary

The anatomy of complex organisms depends on the reliable formation of spatial patterns of gene expression during development. Many factors have to be coordinated to regulate gene expression and stochasticity in these events could undermine pattern formation. One well-studied example of pattern formation is the sequential formation of somites, embryonic segments of the vertebrate body. In this system, a spatial pattern is generated by the movement of a gene expression oscillator along the body. Effective pattern formation requires that neighbouring cells oscillate in synchrony with one another. Genetic experiments have determined that Notch signalling is required for synchrony, leading to the proposal that Notch signalling counteracts noise in this system. However, the source of noise has never been demonstrated. Via mathematical modelling, we explore different sources of noise. We show that the likely source of this noise is the randomness of switching on of key oscillator genes, *her1/7*. Experimental measurements enable us to measure the delay in the timing of activation of the two *her1* gene copies in each cell. This delay allows us to estimate the stochasticity in *her1/7* gene regulation. This delay explains the rate of neighbouring cell desynchronisation in the absence of Notch signalling.

## Introduction

Robust and reproducible generation of patterned tissues is a key feature of metazoan development. Noise in regulatory mechanisms has the potential to disrupt this process. As a result, many regulatory networks have evolved to be robust to noise. One such example is the segmentation of the vertebrate body axis, a remarkably precise process. Segments originate from bilateral blocks of cohesive groups of mesoderm cells, called somites, along the antero-posterior body axis on either side of the neural tube, in a process known as somitogenesis. Ultimately, somites differentiate and give rise to ribs, vertebrae and skeletal muscles of the body. The presomitic mesoderm (PSM), a region of undifferentiated tissue at the posterior of the embryo is the source of newly formed somites. FGF and Wnt are produced in the tailbud and are thought to define the extent of the PSM by maintaining cells in an active, plastic state within range of their signalling. As the embryo grows caudally, cells at the anterior of the PSM continuously emerge and move out of range of these posterior signals. In doing so, they begin differentiation and break up into somites separated by clefts or somite boundaries via a process known as the wavefront of maturation [1–8]. A molecular oscillator, known as the segmentation clock, defines the periodic spacing of the boundaries between successive somites [9]. This segmentation clock involves the regular coordinated cycles of production and degradation of transcripts of certain genes in the tail end of the embryo. During each such cycle, one additional somite is formed as another set of cells emerge from the PSM. It is the cyclic behaviour of the segmentation clock that goes on to establish the segmental pattern of the vertebrae body. This segmentation clock runs at fastest speed in the posterior section of the PSM and it is here that the periodicity of somite formation is determined [9–16]. As cells overflow out of the PSM, they stop oscillating [17], switch on expression of further genes and become arrested in their current state before beginning differentiation [18]. Hence, we observe that the spatially periodic pattern of gene expression is a consequence of the temporal oscillation of gene expression in the PSM.

**Fig 1:**
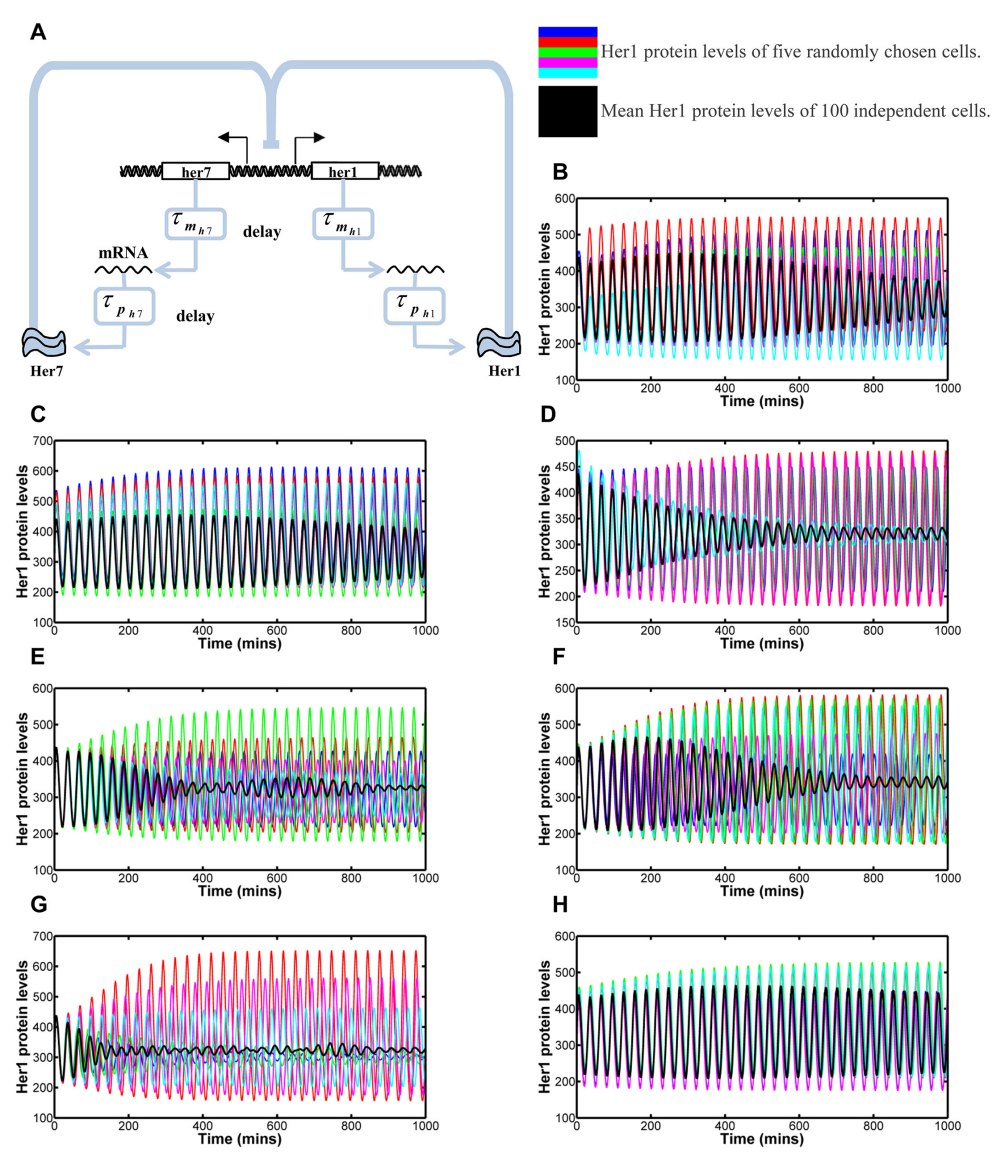
Modelling the effects of inter-cellular variability in transcription, translation and degradation rates and delays. 1A: Her1/7 feedback loop in which Her1/7 protein inhibits expression of *her1/7* genes. There is a delay in transcription of $\tau_{m_{h1/7}}$ and a delay in translation of $\tau_{p_{h1/7}}.$ Stimulation is given by → and inhibition by ⊣. 1B-1H: The oscillating Her1 population levels for five independent cells in the system (blue, red, green, magenta and cyan) and overlaid mean population level of all 100 cells in the system (black) versus time for variability in reaction rate, delay and number of Hes6 molecules. The mean population levels reflect how synchronous the oscillations of neighbouring cells are in addition to the amplitude of oscillation of the individual cells. Inter-cellular variability is distributed as a Gaussian distribution with mean, *μ*, and standard deviation, *σ*, i.e. as *N*(*μ*, *σ* ^2^). The mean value is given by the fitted values for each parameter (see S2 Text) with the maximum variation possible before qualitative changes in oscillatory behaviour occur. Quantification methods for desynchronisation can be found in Methods and S3 Fig. 1B: Variability in transcription rate, *α*_*h*_, distributed as *α*_*h*_ ∼ *N*(33,9^2^). The system is very robust to changes in transcription rate. Cells are still oscillating in synchrony after 30 oscillations. 1C: Variability in translation rate, *β*_*h*_, distributed as *β*_*h*_ ∼ *N*(9.2, 1.5^2^). The system undergoes severe damping when translation rate falls below 4.6 min^-1^. Cells are still oscillating in synchrony after 30 oscillations. 1D: Variability in degradation rate, *λ*_*h*_, distributed as *λ*_*h*_ ∼ *N*(0.23, 0.025^2^). The system undergoes severe damping when degradation rate falls below 0.15 min^-1^. Cell clocks desynchronise in 20–21 oscillations. 1E: Variability in transcription delay, $\tau_{m_{h1/7}},$ for both *her1* and *her7*, distributed as $\tau_{m_{h1/7}}∼N\left(7,0.5^{2}\right)$. Damping of oscillation on individual cells occurs for values below 5 min. Cell clocks desynchronise in 11–12 oscillations. 1F: Variability in translation delay, $\tau_{p_{h1}}$ and $\tau_{p_{h7}}$ for *her1* and *her7* respectively, distributed as $\tau_{p_{h1}}∼N\left(1.1,0.3^{2}\right)$ and $\tau_{p_{h7}}∼N\left(0.7,0.2^{2}\right)$. Cell clocks desynchronise in 20–21 oscillations. 1G: Variability in transcription delay, $\tau_{m_{h1/7}},$ for both *her1* and *her7*, distributed as Generalised Pareto distributions with parameters, location of 5.84, scale of 1.10 and shape of 0.05, resulting in expected values 7 and variances 1.5. In this instance a small number of cells have much increased transcription delay, resulting in the increased variance for the sample. Cell clocks desynchronise in 14–15 oscillations. 1H: Variability in cellular numbers of Hes6 molecules, *p*_*h*6_, distributed as *p*_*h*6_ ∼ *N*(100, 25^2^). Cells are still oscillating in synchrony after 33 oscillations. Inter-cellular variability in the reaction rate and delay constants and number of Hes6 molecules is not of the right magnitude to explain the desynchronisation of oscillation in Notch mutants.

Oscillating genes in zebrafish that are regulated by Notch include *her1* and *her7*, which encode transcriptional repressors; these genes are thought to be the pacemakers of the entire somite clock. When *her1* and *her7* genes are transcribed, there is a delay in the synthesis and export of their mRNAs and, thereby, until the repressor Her1 and Her7 proteins are synthesised. These proteins then accumulate until they autoinhibit *her1* and *her7* transcription [18–24]. Transcription of these genes resumes only when the mRNAs and repressor proteins have degraded. These time delays result in oscillatory *her1* and *her7* mRNA expression within each cell (Fig 1A) [18, 19], and determine the period of oscillation and, ultimately, the size of a somite [18–20, 25, 26].

The cyclic nature of the segmentation clock relies on the internal regulatory dynamics within each cell, which varies from cell to cell and is subject to stochastic effects. Noise is a fundamental part of cell regulation [27–33] and leads to heterogeneity in cellular populations [34]. Hence, it is heavily associated with poor prognosis in disease [35] and allows cells greater flexibility to deal with their environment [36]. For the development of ordered structures such as somites, the system requires the cells to behave as homogeneously as possible. Noise in cell regulation is consequently, an area of research that is increasingly being explored.

All known zebrafish mutations that disrupt PSM oscillations affect components in the Notch signalling pathway [19, 25, 26, 37–47]. When Notch signalling fails, only 5–8 somites form normally, after which somite boundaries become absent or irregular, leading to a failure of segmentation. Jiang et al. (2000) proposed a simple explanation for this phenomenon: all cells in the PSM start out their oscillation synchronously, but the lack of Delta-Notch cell-cell communication leads to a progressive loss of clock synchrony and salt and pepper expression patterns of cycling genes that are required to position the inter-segmental boundaries [17, 47–49]. When blocking Notch with the inhibitor DAPT, for example, approximately 12 somites will form, representing five anterior somites whose boundaries were already determined, and about another seven posterior somites that form before clock desynchronisation is sufficiently severe to prevent boundary formation [48]. Both Notch mutants and chemical disruption of Notch signalling suggest that cells drift out of synchrony in around 5 to 8 oscillations.

To investigate the source of the noise that leads to desynchronisation, we turned to mathematical modelling. We have continued the development of the mathematical model, incorporating *her1/7* and Delta-Notch, begun by Lewis 2003 [20] and most recently expanded by Hanisch et al. 2013 [50]. We introduced probabilistic rate and delay variables for transcription, translation and mRNA and protein degradation before considering the kinetics of transcriptional de-repression and we show that only the latter predicts that cells drift out of synchrony at a rate comparable to Notch mutants. Finally we considered the mathematical modelling of the wildtype situation, such that cells are able to signal to their immediate neighbours via Notch signalling, demonstrating that Notch signalling overrides the levels of noise that we have quantified and keeps neighbouring cells oscillating in synchrony.

## Results

### Inter-cellular variability in the rate and delay constants does not drive rapid drift to asynchrony

To investigate the most likely source of noise, leading to a desynchronisation of Her1/7 oscillations in neighbouring cells, we adapted the mathematical model of [50] (further mathematical models of the system can be found in [51–55]). Within this original model, the Her1/7 oscillator in multiple cells is considered, either with, or in the absence of, Notch signalling. The system is modelled over a lattice of cells that can signal to one another with the inclusion of Notch signalling. mRNA and protein levels within the system are modelled deterministically whilst gene regulation can be considered stochastically or deterministically. Deterministic modelling of mRNA and protein is reasonable, due to the large numbers of molecules involved. Transcription and translation delays are incorporated by making use of delay differential equations (DDEs). Our adaptations to the model focused on incorporating stochasticity and inter-cellular variability into the various mechanisms of the model in order to investigate their effects. We introduced random variables for rate and delay constants and the number of Hes6 molecules to investigate whether desynchronisation of independently oscillating cells is a result of inter-cellular variability in the transcription, translation or degradation rates, the transcription or translation delays or the number of Hes6 molecules. The modelling of stochastic gene regulation was altered significantly to make the mechanisms more realistic and therefore the results more accurate, in order to correctly investigate the effects on the system. In depth information about the model, how it has been adapted, and parameter choices can be found in S1 and S2 Texts. For such analysis we carried out our simulations without Notch signalling and considered 100 independently oscillating cells. Following on from the observations of [17] whereby when somitogenesis begins, cells begin their oscillations in synchrony, cells were modelled with identical initial populations of *her1* and *her7* mRNA and Her1 and Her7 protein and the same number of *her1* and *her7* genes switched on (when considering the gene regulatory level).

To determine if inter-cellular variability in the transcription, translation or degradation rates or transcription or translation delay could be the source of noise that causes cells to drift out of synchrony, we considered a purely deterministic model for *her1/7* mRNA and protein regulation. Parameter space exists that does not lead to oscillation (for example, very short delays leads to no oscillation), this area of parameter space was excluded from further consideration. We introduced inter-cellular variability by selecting the relevant parameter value from a Gaussian distribution centred at the fitted value for that parameter. We aimed to maximise the standard deviation of these distributions, without affecting the qualitative behaviour of the oscillations such that some cells no longer exhibited oscillation.

Fig 1B–1D illustrates that this simple model, incorporating just the *her*1/7 negative feedback loop, is capable of generating oscillations within the system and demonstrate the effect of inter-cellular variability in the transcription, translation and degradation rates respectively. The coloured lines give the Her1 protein levels for five randomly selected cells whilst the black line gives the mean Her1 protein levels for all 100 cells. The amplitude of oscillation of the mean reflects the amplitude in the individual cells in addition to the degree to which neighbouring cells are in synchrony. Altering the transcription, translation or degradation rate of a molecule primarily affects the amplitude of oscillation of that molecule whilst effects on period of oscillation are generally small (S1A–S1C Fig). Each individual cell has a different, randomly generated, rate constant affecting the oscillations in that single cell, as demonstrated by the smooth behaviour of the amplitude and period curves of each cell. Thus, we demonstrate that individual cells still oscillate almost in synchrony, just with amplitudes independent of each other. In each case, there is a small damping effect on the mean amplitude of all cells. Random degradation results in the greatest damping but, this is a reflection, primarily, of the amplitude of oscillation decaying to a smaller magnitude in cells with a low degradation term (low degradation of the inhibitory proteins results in lower levels of gene expression). Quantitatively, we defined the point at which desynchronisation of neighbouring cells was reached based on how the sample distribution of Her1 protein levels in the 100 cells changed over time (see Methods). Inter-cellular variability in transcription and translation rates results in the cells still oscillating in synchrony with one another after 30 oscillations, whilst cells desynchronise in 20–21 oscillations in the case of variability in degradation. The system is very robust to the effects of inter-cellular variability in transcription, translation and degradation rates.

Following on from this, we investigated the effects of inter-cellular variability in transcription and translation delay. Transcriptional and translational delays in the model, which incorporate such things as splicing and export time to the cytosol, determine the period of oscillations in the system [20] and also affect amplitude of oscillation (S1D and S1E Fig). Cells drift out of synchrony in 11–12 clock cycles due to variability in transcription delay (Fig 1E)and 20–21 oscillations in the presence of variability in translation delay (Fig 1F). The slow drift out of synchrony is due to each individual cell having its own period of oscillation that tends to a constant value that is independent of other cells (S1D and S1E Fig).

We observed that the maximum amount of variation in transcription and translation delay allowable in our model is lower than that quantified experimentally [18]. This suggests that such delays may be asymmetrically distributed such that the effect of increased variance would still result in no cells having such low delays that oscillations cease and a small number of cells having very long delay times. To consider the effect of an increased variance and skew in the distributions of delay we considered the heavily skewed Generalised Pareto distribution. Increasing the variance of transcription delay to be comparable to experimental data [18] resulted in a small effect on desynchronisation rates (Fig 1G) with cells desynchronising in 14–15 oscillations in this example, as the increase in variation was due, predominantly, to a small number of cells oscillating with much longer periods.

Finally, within our model, Her1 functions as a homodimer and Her7 as a heterodimer with Hes6, with the number of Hes6 molecules assumed constant in each cell as in [50]. We thus considered the effects of inter-cellular variability in the number of Hes6 molecules. Variation in this term affects the amplitude of oscillation with only marginal effects on period of oscillation (S1F Fig) resulting in cells still oscillating in very close synchrony after 33 oscillations (Fig 1H).

Inter-cellular variability in the reaction rate and delay constants and number of Hes6 molecules causes a desynchronisation in 11–12 oscillation cycles at the minimum. This would mean that on blockade of Notch signalling with DAPT, a further 16-17 somites would form correctly (5 anterior somites and the 11-12 posterior somites prior to desynchronisation) and is therefore not consistent with the experimental results in Notch deficient embryos. The speed of desynchronisation is determined by the magnitude of variation between cells. Inter-cellular variability has been considered at the maximum possible, using Gaussian distributions, such that we still maintain oscillation in our model. Reducing the inter-cellular variability from the levels described here decreases the speed of desynchronisation. Only severe changes in parameter values resulting in individual cells ceasing oscillation will cause the mean amplitude of all cell oscillations to decay to zero at the correct rate. This decay would therefore not illustrate the desynchronisation of neighbouring cells as is argued to occur in [17, 20, 56, 57], but the fact that some cells exhibit damped oscillation and some go on to stop oscillating entirely. We therefore ruled out inter-cellular variability in rate and delay constants and cellular numbers of Hes6 molecules as being the main driver of desynchronisation. Hence, we turned to investigate the effects of noise in the stochastic nature of binding between molecules in the system.

### Stochastic gene regulation can explain the rate of clock desynchronisation when Notch signalling fails

**Fig 2:**
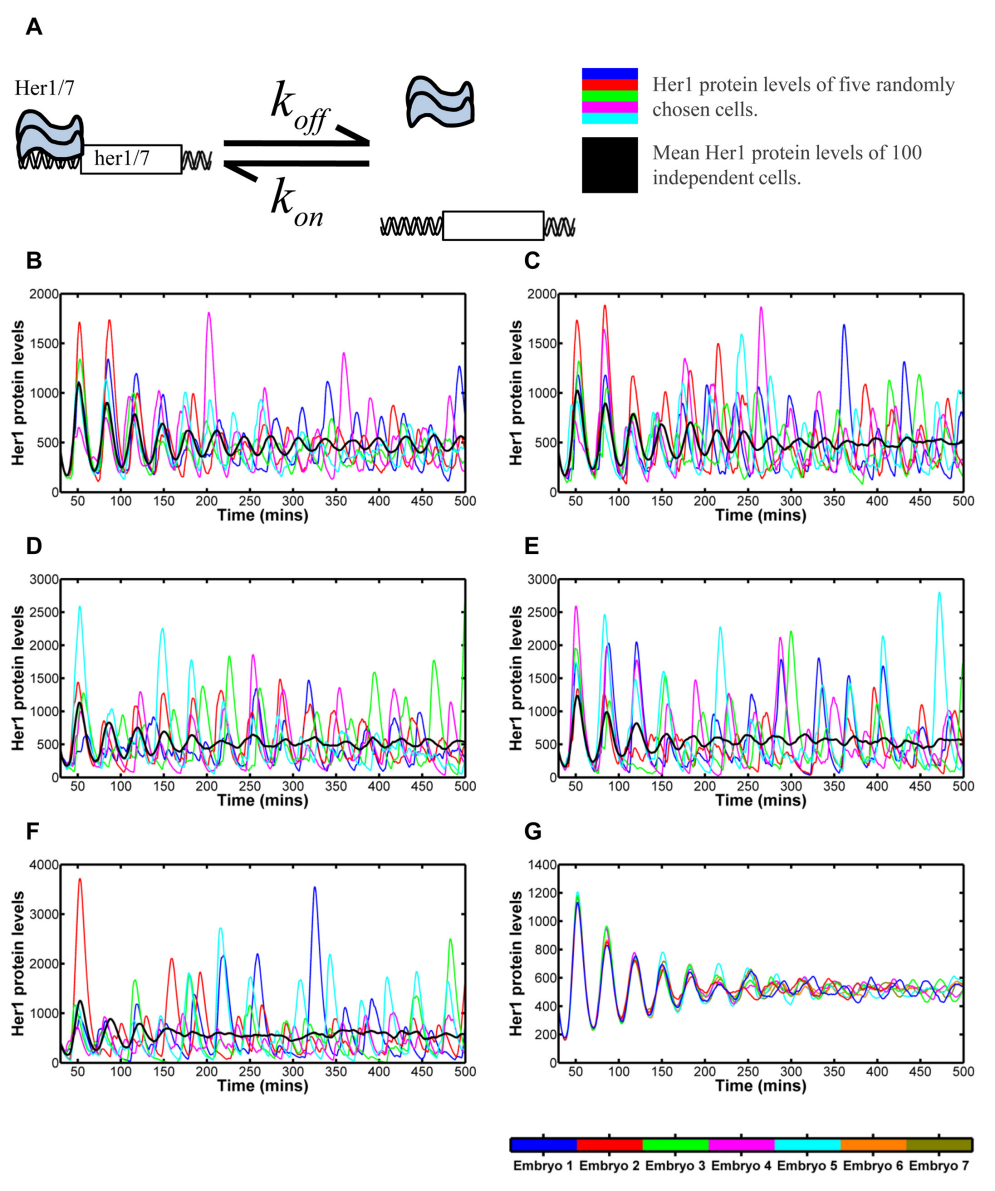
Modelling the effects of stochastic dissociation. 2A: Reaction kinetics of Her1/7 proteins binding to the inhibitory sites on *her1/7* DNA to switch off expression of *her1/7*. 2B-F: The oscillating Her1 population levels for five independent cells in the system (blue, red, green, magenta and cyan) and overlaid mean population level of all 100 cells in the system (black) versus time for differing $k_{off_{Her1/7}}$ values relating to the her1/7 inhibitory reaction described in 2A. The mean population levels of all 100 cells primarily reflect how synchronous the oscillations of neighbouring cells are. Quantification methods for desynchronisation can be found in Methods and S3 Fig. 2B: $k_{off_{Her1/7}}=1$ min^-1^. Cell clocks are still in synchrony after 14 oscillations. 2C: $k_{off_{Her1/7}}=1/2$ min^-1^. Cell clocks desynchronise in 11–12 oscillations. 2D: $k_{off_{Her1/7}}=1/3$ min^-1^. Cell clocks desynchronise in 6–7 oscillations. 2E: $k_{off_{Her1/7}}=1/4$ min^-1^. Cell clocks desynchronise in 5–6 oscillations. 2F: $k_{off_{Her1/7}}=1/6$ min^-1^. Cell clocks desynchronise in 2–3 oscillations. 2G: Mean population levels of 100 cells for seven different simulations, for $k_{off_{Her1/7}}=1/3$ min^-1^. There is variability in the embryos and cell clocks desynchronise in 6–8 oscillations. The figure demonstrates that noisy gene regulation can drive desynchronisation in Notch mutants.

A significant source of noise in reaction kinetics arises from the randomness of which molecules associate or dissociate from one another and when these reactions occur. Chemical reactions are inherently noisy with population levels changing by discrete amounts in a manner that is not wholly predictable. Therefore, deterministic modelling is generally accurate only when population levels are so large that random fluctuations can be ignored [58–61]. In the analysis above, only rate and delay constants were fitted randomly, the chemical reactions themselves were modelled deterministically. For modelling of mRNA and protein behaviours, this is reasonable due to the large numbers of molecules in each cell. However, cells in G1 phase of the cell division cycle will have only two copies of the DNA encoding for Her1 and Her7. Hence, noise in the chemical kinetics will be dominant at the level of gene regulation, i.e. switching genes on or off. Stochastic gene regulation is well studied with a wealth of papers carrying out experimental [27–29, 33, 36, 62] and mathematical [63–66] analysis of the stochasticity. Indeed, Horikawa et al. [67] have previously noted that this noisy gene regulation, alongside the stochastic nature of cell division, is responsible for the variation in oscillation phases in neighbouring cells, in wildtype embryos. Therefore, we investigated if desynchronisation could be caused by the stochasticity of the binding and dissociation of gene regulatory proteins Her1 and Her7 to and from their sites on *her1* and *her7* DNA (Fig 2A).

To analyse the stochastic effects of gene regulation on the system, we combined stochastic modelling of gene regulation with a deterministic modelling of the mRNA and protein levels. We significantly adapted previous hybrid models [20, 50] to provide the gene regulatory level with a firmer physical basis in order to model the stochasticity as rigorously as possible. To do so, we developed a hybridised model of deterministic DDEs and The Gillespie Algorithm such that both the next gene regulatory reaction to occur and the time that this reaction takes to occur are random (see Discussion, Methods, S1 Text).

In Fig 2, we demonstrate that stochastic gene regulation can cause Her1/7 oscillations in neighbouring cells to drift out of synchrony when the cells are independent [20]. We varied the dissociation parameter, $k_{off_{Her1/7}},$ between 1 and 1/6 min^-1^, values being the same order of magnitude as those selected in [50], and observed its effects on desynchronisation of independent Her1/7 oscillations (Fig 2B–2F). Within each individual cell, the mean amplitude and mean period of oscillation remains fairly constant, closely resembling the deterministic cases (S2A–S2C Fig). Random fluctuations around the mean period build up over time (S2D Fig) and cause cells to drift out of synchrony from one another. Thus, the mean amplitude over all cells decaying to zero (black line, Fig 2) illustrates purely the desynchronisation of neighbouring cells.

**Fig 3:**
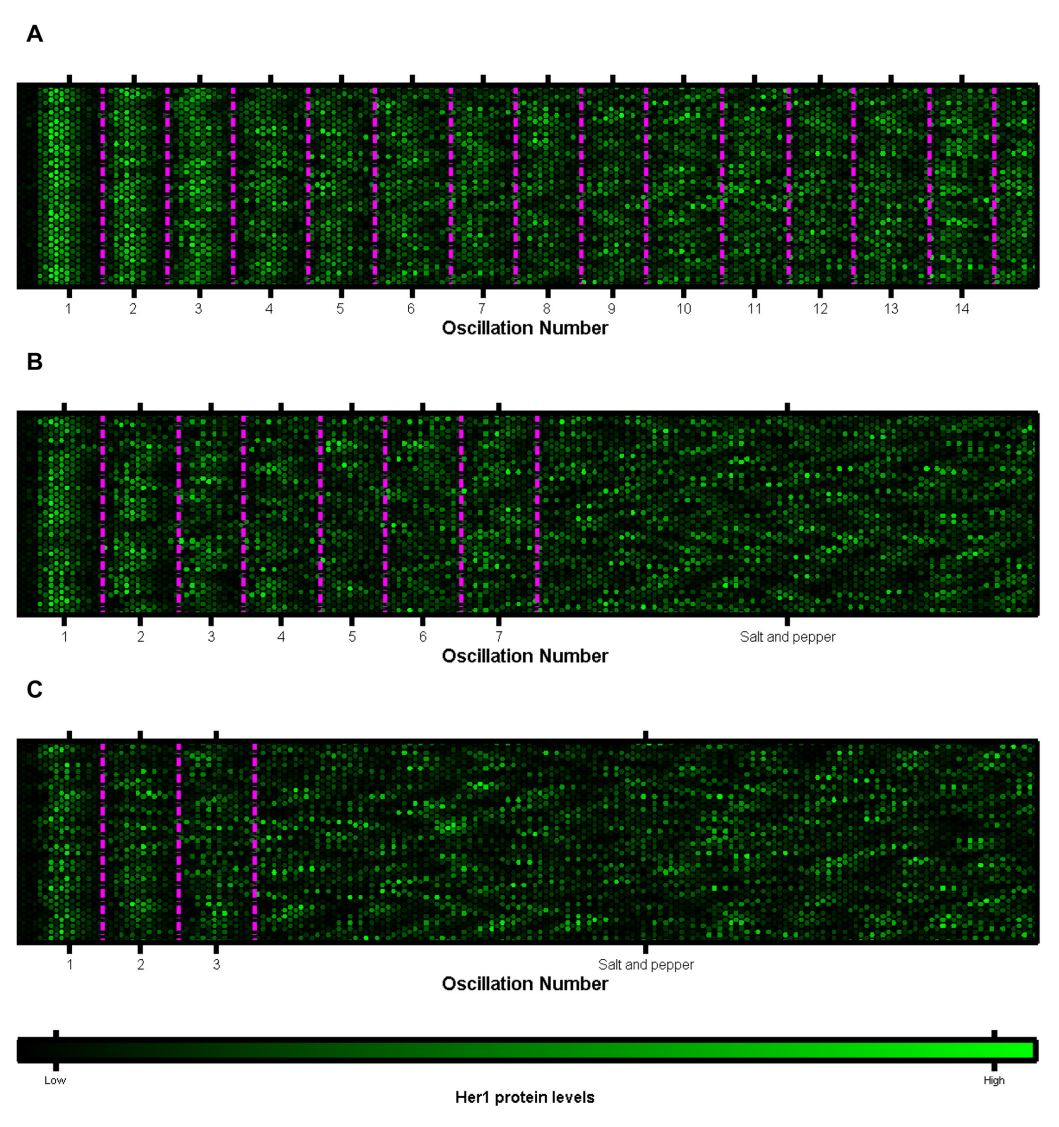
Plots of multiple cells oscillatory clocks versus time. Each lattice is a single cell. Each five minute interval is marked by two columns of 32 cells. The mean phases of oscillation over all cells are marked by the magenta lines. The data is that of Fig 2. A: $k_{off_{Her1/7}}=1$ min^-1^. The cells remain in synchrony and no salt and pepper pattern is generated. B: $k_{off_{Her1/7}}=1/3$ min^-1^. The cells gradually drift out of synchrony to a salt and pepper pattern over 6–7 oscillation cycles. C: $k_{off_{Her1/7}}=1/6$ min^-1^. Cell oscillations quickly desynchronise and tend to a salt and pepper pattern in 2–3 oscillation cycles. The figures demonstrate that, gradually, the number of cells oscillating in phase, within the magenta periods, decreases as noisy gene regulation causes the cell clocks to gradually drift out of synchrony from one another. The result is a salt and pepper pattern. As this salt and pepper pattern is reached, somites will not form correctly. (Compare to the wildtype case of Fig 5E.)

We find that cells take at least 14 oscillations to lose synchrony for $k_{off_{Her1/7}}=1$ min^-1^ (Fig 2B), 11–12 oscillations for $k_{off_{Her1/7}}=1/2$ min^-1^ (Fig 2C), 6–7 oscillations for $k_{off_{Her1/7}}=1/3$ min^-1^ (Fig 2D), 5–6 oscillations for $k_{off_{Her1/7}}=1/4$ min^-1^ (Fig 2E) and 2–3 oscillations for $k_{off_{Her1/7}}=1/6$ min^-1^ (Fig 2F). We observe that by decreasing the value of this parameter, $k_{off_{Her1/7}},$ we increase the magnitude of noise in gene regulation and increase the speed at which cells lose synchrony from one another (S2D Fig). This is a consequence, in part, on the fact that the amount of Her1/7 produced in a cell is dependent on the time that each gene remains active for. The timing of the stochastic association/dissociation reactions is distributed exponentially with variance proportional to $1/k_{off_{Her1/7}}{}^{2}.$ Hence, as $k_{off_{Her1/7}}$ decreases in magnitude, the variability in timing of reaction events increases and this increases variability in Her1/7 populations between cells. Greater inter-cellular variability in protein populations between cells goes on to further increase the variability in timing of reaction events.

Despite the mean Her1 levels over all cells gradually decreasing to zero, the peaks and troughs of this inter-cellular mean still occur at the correct points in time, corresponding to a cellular period of oscillation of approximately 30 minutes. When cells oscillate independently from one another, individual cell oscillations continue to exhibit the correct amplitude for their location but, since cells are out of synchrony, a random mixture of cells in different phases of oscillation cycles is produced [17] as seen by the coloured lines of individual cells drifting out of phase with one another as time progresses in Fig 2 and in agreement with the dynamic *in vivo* analysis of [56, 57]. The result of this asynchrony is a salt and pepper expression of *her1* and *her7* in the PSM [20]. In S4 Fig and S1–S3 Movies we present the cells as a lattice, with each cell represented by a hexagon with the brightness reflecting the Her1 protein levels. The salt and pepper pattern is reached more quickly, the greater the level of stochasticity in gene regulation. By considering Her1 protein levels in 64 cells at five minute intervals (given by each double column of 32 hexagons) in Fig 3, we illustrate how the desynchronisation of cell clocks can go on to determine the structure of future somites. As the cell clocks approach desynchronisation, the ordered structure of cells (highlighted by the magenta boundaries) becomes more disjointed, ultimately leading to somite boundaries becoming absent or irregular and the failure of somitogenesis.

### Expression onset of the two *her1* gene copies differs by approximately three minutes in a PSM cell

The analyses above imply that noisy gene regulation might drive the desynchronisation of oscillations in PSM cells in the zebrafish, in the absence of Notch. To support this view, we have performed in situ hybridisation to quantify the magnitude of this noise parameter.

**Fig 4:**
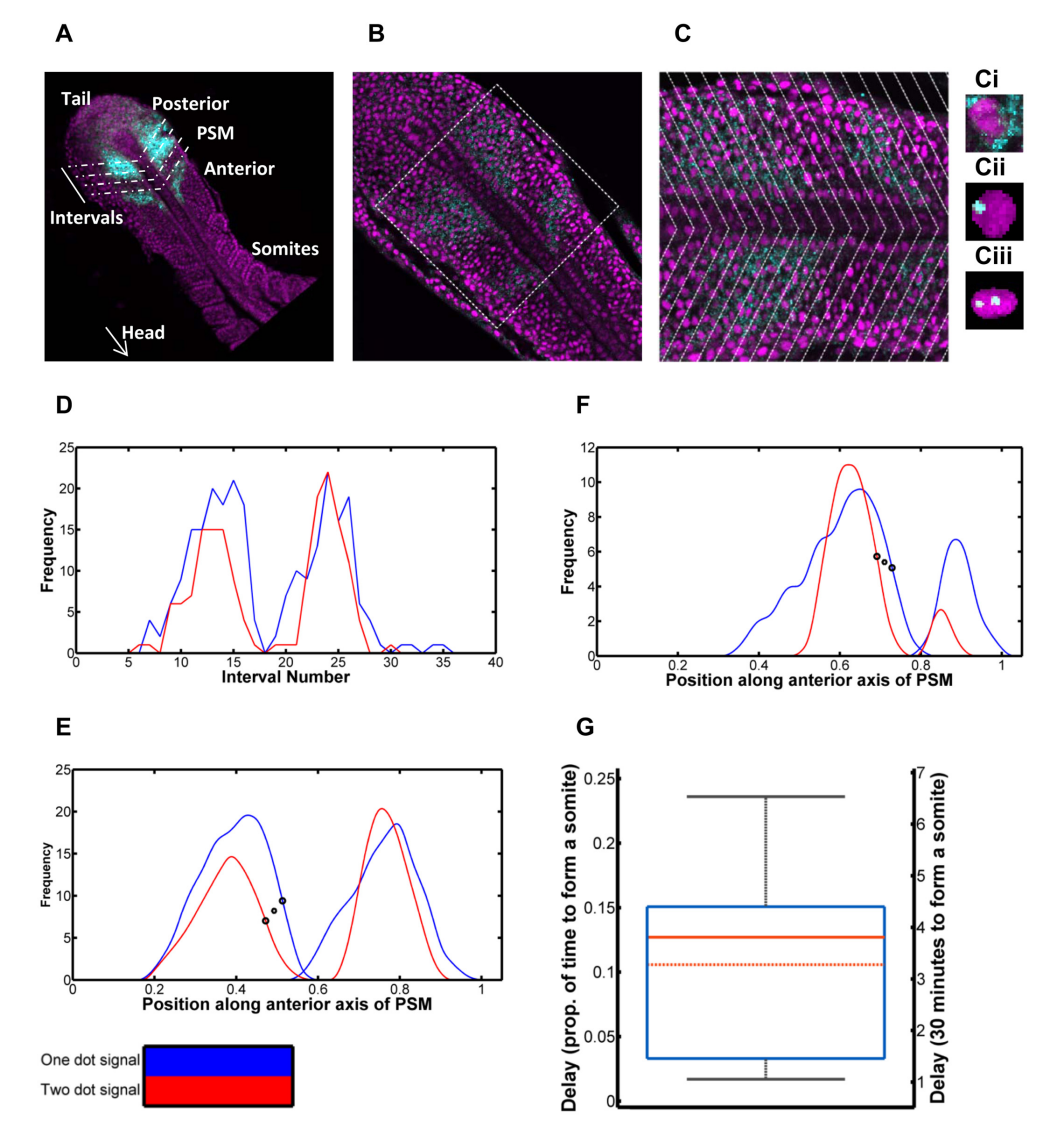
Quantification of delay in expression between *her1* gene copies in a PSM cell. 4A: Schematic of quantification. Oscillatory gene expression in the posterior of the PSM is shown in cyan. The PSM is divided into intervals in order to quantify this gene expression, spatially. 4B: A single slice of a zebrafish embryo with nuclei in magenta and *her1* mRNA in cyan. 4C: The zoomed in square of 4B to closer demonstrate the cytoplasmic *her1* mRNA molecules and *her1* transcripts in the course of synthesis. In both figures, the cyan stripes of *her1* mRNA are apparent. Intervals are overlaid, based on the gradient of the *her1* mRNA waves, and the frequency of nuclei with one *her1* transcript in the course of synthesis and those with two is quantified per interval. 4Ci: A cell containing cytoplasmic *her1* mRNA molecules. 4Cii: A cell with one *her1* gene copy expressed. 4Ciii: A cell with two *her1* gene copies expressed. 4D: Plot of frequency of nuclei with one dot (blue) and frequency with two dots (red) per interval. The two dot signal is delayed behind the one dot signal. 4E: The signals smoothed and the interval scale transformed to distance over the anterior axis (scaled from zero to one). The large black circles demonstrate the inflection points used to calculate the delay. This specific delay is selected as the increase in frequency of active *her1* genes is a reflection of the repressing Her1/7 protein dissociating from the gene. The smaller symbol, between the two circles, gives the x coordinate used for further calculation in each case. 4F: Further example of smoothed dot count signals for an additional embryo. 4G: Box and whisker plot of delay between one and two dot signals, as a proportion of time to make one somite (left axis) and in minutes assuming the time to make one somite is thirty minutes (right axis). The minimum and maximum are given by the whiskers, the lower quartile and upper quartile by the box. The solid red line gives the median and the dot-dash red line gives the mean. The predicted delay in expression between the two *her1* gene copies in a cell is three minutes. The mathematical model predicts cells will desynchronise in 6–8 oscillations when inserting the dissociation rate, $k_{off_{Her1/7}}=1/3$ min^-1^, corresponding to this delay.

During somitogenesis, oscillations of gene expression gradually reduce in frequency as cells move through the PSM, until they are halted completely in the anterior; the further anterior cells lie in the PSM, the more their phase is retarded (see Fig 4A for a schematic of where this occurs in the embryo). As a consequence, phase differences of the clock are manifest as spatial offsets in the PSM, which can be used to calculate the timing of delay (see [18] for a more detailed description of the methodology). Thus, Giudicelli et al. [18] previously measured a 4 minute delay between the onset of *her1* transcription and the appearance of *her1* cytoplasmic mRNA.

The stochastic nature of gene regulation causes a temporal delay between the expression of the first *her1* gene in a cell and the second. Stripes of cells expressing a single copy of the gene lie slightly anterior to cells expressing two copies of the gene [18].We carried out fluorescent in situ hybridisation (FISH) on wildtype embryos to detect sites of active transcription of *her1*, as seen in Fig 4B and 4C. The periodic stripes of *her1* mRNA are visible in cyan, in both figures. Some cells contained only cytoplasmic *her1* mRNA molecules (Fig 4Ci) whilst others contained *her1* transcripts in the course of synthesis; being evident as intense dots within the nucleus. The vast majority of transcripts within each cell numbered zero to two, consistent with the number of gene copies in G1 cells.

We therefore quantified the spatial distance between the stripes of cells expressing one *her1* gene copy (Fig 4Cii) and two gene copies (Fig 4Ciii). For each embryo, we carried out an image analysis in MATLAB (Methods, S3 Text and S5 Fig), dividing each 3D stack into intervals along the anteroposterior axis such that we could count the number of nuclei with one and two dots in each interval (Fig 4A and 4C) and quantify the frequency of cells expressing one gene copy and two gene copies versus spatial position (Fig 4D–4F). The temporal delay can be calculated from this spatial displacement by considering how the clock rate changes with cell position and the way the tailbud extends caudally due to proliferation (Methods and [18]).

Assuming that the time to make one somite is 30 minutes (see [68] for details of temperature dependence of somitogenesis period) the mean delay is estimated to be 3.4 minutes (n = 11, 95% CI 2.5 mins ≤ *μ* ≤ 4.3 mins). From the sample distribution (Fig 4G, S1 and S2 Tables), we estimated the population statistics using non-parametric bootstrapping (see Methods) due to the sample-size being small and the underlying probability distribution being unknown and possibly non-Gaussian. This provides us with a measure of the delay in expression between the two gene copies in a cell, caused by noisy gene expression, of 3 minutes. This delay most likely corresponds to the stochastic de-repression of the *her1* gene (and in subsequent initiation of transcription) and provides us an estimate for the magnitude of noise in this gene regulation.

### Modelling the measured stochastic delay predicts the loss of clock synchrony in 6–8 clock cycles in the absence of Notch signalling

The measured 3 minute delay corresponds to the dissociation half-life of the inhibitory protein from the *her1/7* DNA $\left(k_{off_{Her1/7}}\right)$ of 1/3 min^-1^. Fig 2D shows that, when $k_{off_{Her1/7}}=1/3$ min^-1^, the cells drift out of synchrony after approximately 6–8 oscillations. There is, of course, also variability in rate of desynchronisation between different embryos, due to stochastic gene regulation (Fig 2G). Although desynchronisation is more rapid in some simulations, the general trend is that it takes 6–8 oscillations before cells are no longer in synchrony with one another. Strikingly, this is in agreement with experimental data [17, 48]. Consequently, the measured dissociation rate of Her1/7 from their binding sites on *her1/7* DNA is sufficient to explain the *in vivo* rate of clock desynchronisation, suggesting that the stochasticity of *her1/7* de-repression is, most likely, the key noise parameter that drives loss of synchrony in the absence of inter-cellular Notch signalling.

### Notch signalling can override stochastic effects and force cells into synchrony

**Fig 5:**
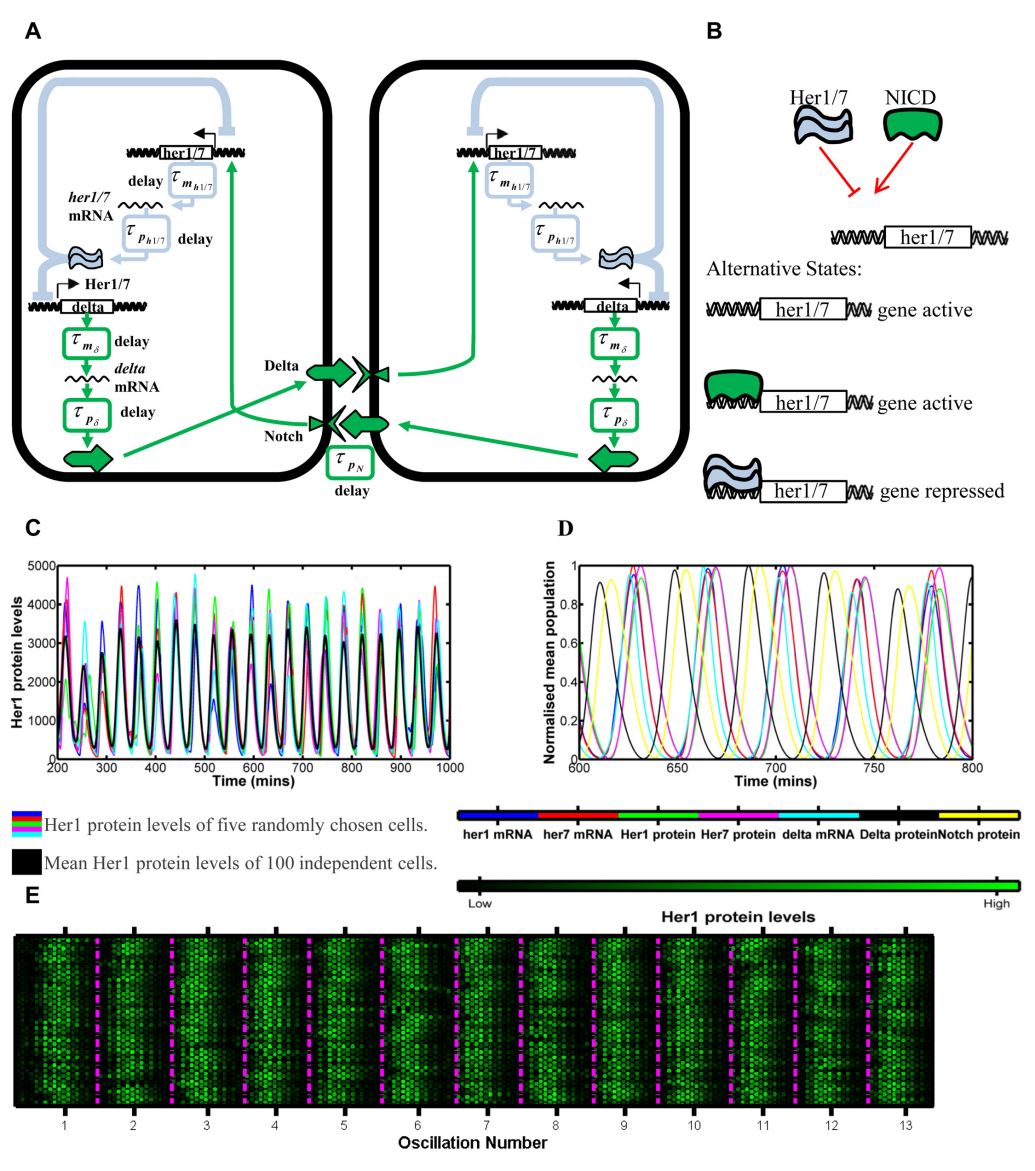
Modelling the effects of Notch signalling on the synchrony of neighbouring cell oscillations. 5A: Notch signalling network between neighbouring cells. The light blue section corresponds to the intra-cellular *her1/7* feedback loop of Fig 1A. The green section corresponds to Notch inter-cellular signalling. Her1/7 protein inhibits expression of *delta* in addition to *her1/7*. Transcription and translation of *delta* occurs with respective delays $\tau_{m_{\delta }}$ and $\tau_{p_{\delta }}.$ Delta activates Notch in the neighbouring cell and NICD is produced with delay, $\tau_{p_{N}}.$ When Notch binds to the *her1/7* genes this leaves them in an active state, influencing the *her1/7* intra-cellular feedback loop. 5B: Competitive binding reaction kinetics of Her1/7 and Notch proteins to the sites on *her1/7* DNA. When the *her1/7* gene is free or bound to NICD then the gene is active. When Her1/7 binds to the *her1/7* gene then expression of the gene is inhibited. It is these reaction kinetics that we model with a modified Gillespie Algorithm. 5C: Plot of oscillating Her1 levels versus time when Notch signalling has been incorporated into the model. The coloured lines correspond to five randomly selected individual cells; the black line corresponds to the mean of all 64 cells. The cells oscillate in synchrony. 5D: The normalised mean population levels of *her1* mRNA, *her7* mRNA, Her1 protein, Her7 protein, *delta* mRNA, Delta protein and Notch protein demonstrating the phase of each population. Notch is almost totally out of phase with Her1/7 so will be high when Her1/7 is low and vice versa. 5E:Plots of multiple cells oscillatory clocks versus time (compare to Fig 3). Each lattice is a single cell. Each five minute interval is marked by two columns of 32 cells. The mean phases of oscillation over all cells are marked by the magenta lines. The cells remain in synchrony throughout and generate well defined somites. The modelling demonstrates that Notch signalling is able to override the effects of stochastic gene regulation and keep neighbouring cell clocks oscillating in synchrony.

In the posterior of the PSM, the major function of Notch is to keep the oscillations of neighbouring cells in synchrony [17, 18, 20, 48, 57, 69]. The *her1/7* negative feedback loop, in which *her1/7* products inhibit their own expression, also regulates the expression of *deltaC*, a Notch ligand, triggering oscillations of this protein and thereby causing periodic Notch activation in neighbouring cells. This, in turn, stimulates expression of *her1* and *her7*. This feedback mechanism, first proposed by Lewis [20], explains how cells are able to provide information to their neighbours such that they can adjust their internal clocks (Fig 5A where the blue section refers to the intra-cellular *her1/7* circuit whilst the green section refers to the inter-cellular Notch signalling component).

To investigate whether oscillating gene expression is indeed robust to the effects of stochastic gene regulation, due to the coupling effects that Notch provides, we added Notch signalling to our mathematical model. We modelled the reactions between Notch intra-cellular domain (NICD) and *her1/7* genes stochastically, however, the inter-cellular signalling was modelled purely deterministically. *delta* mRNA and protein, and NICD were incorporated into the model with delay again manifesting in transcription and translation of *delta* and in activation of Notch. It is believed that NICD and Her1/7 compete [48] to bind to the regulatory DNA of *her1/7* in order to maintain synchrony between cells (see Fig 5B) and it is here where stochastic behaviour again comes in to play. For instance, if Her1/7 binds to the regulatory site of *her1/7*, this would inhibit the expression of *her1/7*. However, if NICD is already bound to the regulatory site then this prevents the inhibition from occurring. We considered a lattice of 64 cells with periodic boundary conditions. The dissociation rate of Her1/7 from its site of *her1/7* DNA, $k_{off_{Her1/7}},$ was selected as 1/3 min^-1^. At initialisation, the number of gene copies bound to Her1/7 and NICD and number of mRNA and proteins in each cell were determined randomly in order to demonstrate the effects of Notch signalling on desynchronised neighbouring cell oscillations.

Our mathematical model demonstrates that the inclusion of Notch signalling can override stochastic effects that would otherwise cause loss of clock synchrony (see the uniform expression of cells in S4 Movie). Fig 5C shows the Her1 oscillations for five Notch-competent cells (coloured lines) with the mean Her1 levels (black line). The five cells show similar oscillation periods and phases (although with variable amplitude), showing that Notch signalling imposes synchrony on the population (S6 Fig). Fig 5D shows the normalised mean levels of each population versus time to demonstrate how each population oscillation is out of phase from the others. Due to the delays in the system, levels of Notch are almost totally out of phase with levels of Her1/7, meaning that when Her1/7 levels are low, levels of Notch are high and *her1/7* gene activation is more likely and vice versa. Fig 5E illustrates that, with the inclusion of Notch signalling, the boundaries of future somites will be well defined. Further model validation at the gene regulatory level can be found in S4 Text.

## Discussion

In the absence of Notch signalling, the clocks of neighbouring cells desynchronise in 5–8 clock cycles. We have provided a possible mechanism for the source of this noise: the stochastic nature of the binding of Her1/7 protein to inhibitory sites on *her1/7* DNA. The stochastic nature of dissociation of Her1/7 from its target sites leads to a temporal delay between the expression of the first *her1* gene copy in a cell and the second. We used in situ hybridisation to quantify this delay and measured a delay of approximately three minutes. The corresponding dissociation rate, 1/3 min^-1^, inserted into our mathematical model predicts that the oscillations of Her1/7 desynchronise in 6–8 oscillations, in broad agreement with the observed effects of losing Notch-mediated synchronisation. Our results further validate the ideas and predictions made in Lewis. 2003 [20] and later publications that mathematically describe the oscillatory mechanism of *her1/7* and Notch signalling’s role in keeping neighbouring cell clocks in synchrony. In addition, it was suggested that stochastic gene regulation of *her1/7* was a likely driver of desynchronisation in the absence of Notch signalling [20, 49, 50] and the results herein provide robust quantification of such arguments.

### Delay in *her1* gene expression is a consequence of stochasticity in the repressor/DNA dissociation

In a single cell, the two *her1* genes are in an identical nuclear environment, exposed to the same repressor concentrations. Therefore, the difference in timing in expression between the first *her1* gene copy and the second, of approximately 3 minutes, is most likely a consequence of the stochastic dissociation of Her1/7 repressor proteins from the *her1* target genes (and subsequent noise in initiation of transcription). Gene expression is repressed in the protein bound state and therefore, the key noise parameter, the half-life for dissociation of the inhibitory protein from the DNA, can be estimated from the delay in expression between the two gene copies. This half-life for dissociation reflects the mean time taken for the protein to become dissociated from the gene and the inhibited gene to begin expression again. In Fig 4, the number of *her1* transcripts in the course of synthesis per cell is a reflection of the number of genes without repressors bound. Further stochasticity in gene regulation will also be present in, for example, the recruitment of the core transcriptional machinery and this is currently not explicitly included in our mathematical model (its contribution to delay in expression between gene copies is effectively incorporated into the magnitude of our dissociation rate, $k_{off_{Her1/7}}).$ In S7 Fig we compare the delay in *her1* gene expression between our experimental and simulated data. We note that although the mathematical model has captured the average delay very well, it introduces far less variability in delay than in our experimental data. Although the increased variation in the experimental data will in part be caused by experimental measurement, we note that this could also be a consequence of further stochasticity in transcription initiation. The introduction of inter-cellular variability does not significantly alter the distributions of delay, giving further weight to the argument that delay in gene expression is likely to be a consequence of stochasticity in repressor/DNA dissociation.

### Stochasticity in gene regulation dominates over stochasticity in mRNA and protein reactions, due to the low numbers of molecules involved

Stochastic modelling of chemical reaction kinetics is generally carried out either using the chemical master equation [70–72] the solution of which provides the time-evolving probability distribution of molecular populations or The Gillespie Algorithm, which makes ‘exact’ numerical calculations, within the framework of stochastic formulation using a Monte Carlo procedure, to simulate the time evolution of the system [73, 74]. For macroscopic processes, by making use of The Central Limit Theorem, the solution to the master equation can be approximated by a multivariate Gaussian distribution [59–61]. With such solutions, for a system of size *N*, the ratio of the standard deviation to the expected value will be of order *N* ^−1/2^ [61]. Thus, as the size of the system increases, the relative magnitude of variability decreases. Kurtz (1972) demonstrates that if the deterministic equations have a unique stable solution then the deterministic model will be the infinite system-size limit of the stochastic model [58]. Therefore, the smaller the populations, the more dominant noise will become. In a single cell, stochasticity in chemical reaction kinetics will be dominant at the gene regulatory level due to there being only two gene copies per cell of *her1/7* and large numbers of mRNA and protein.

### The Notch signalling pathway must be robust to further stochastic gene regulatory effects in order to keep neighbouring cells oscillating in synchrony

We have demonstrated that Notch signalling is able to override stochastic effects in the gene regulatory system and keep the oscillations of neighbouring cells in synchrony with one another. Within the inter-cellular model, we wanted to investigate the effects of noisy gene regulation of *her1/7* on desynchronisation of neighbouring cell clocks in the absence of Notch signalling and hence, this is the only gene regulation that we modelled stochastically. Noise will also be present in for example, stochastic gene regulation of *delta*. An analysis of noise elsewhere in the Notch signalling pathway would further inform us on how noise within the inter-cellular Notch signalling pathway affected its ability to override stochasticity in the transcription initiation of *her1/7* and keep neighbouring cells in synchrony. Such analysis may provide further avenues for research into the effects of stochastic gene regulation in this system.

### The mathematical model incorporated deterministic delay differential equations with the stochastic Gillespie Algorithm

In order to investigate the effects of various sources of noise on the system, we adapted the mathematical model of [50]. Firstly, we adapted the model such that the rate and delay parameters were distributed randomly. This enabled us to rule out inter-cellular variability as being the driver of desynchronisation. Secondly, in order to investigate the effects of stochastic gene regulation on the system, stochastic gene regulation was modelled more realistically by hybridising delay differential equations modelling mRNA and protein with The Gillespie Algorithm modelling the stochastic gene regulation of *her1/7*.

Previous hybrid models, incorporating stochastic gene regulation and deterministic DDEs, included the stochasticity in a format in which each stochastic reaction will occur over a fixed timestep [20, 50]. Thus, the population levels of active genes can change only at each timestep. Such methods have clearly produced valuable results however, in the words of Gillespie, such a method ‘becomes exact in the limit of the timestep tending to zero, but unfortunately the efficiency of the procedure becomes nil in that same limit’. Therefore, modelling the gene regulation with a fixed timestep will average out some of the stochastic effects and result in behaviour intermediate between deterministic and stochastic models. Since we were carrying out an investigation into the effects of noise within our system, we desired to model it as rigorously as possible and so, in our hybrid model, we turned to incorporating the stochasticity by hybridising DDEs with The Gillespie Algorithm. Incorporating the Gillespie Algorithm, for modelling stochastic gene regulation, results in both the next reaction to occur and the time that this reaction takes to occur being made random making the stochastic element in our hybrid model truly random. Both The Gillespie Algorithm and chemical master equation have been previously adapted to directly incorporate delay [63, 65, 66, 75]. However, hybridisations of The Gillespie Algorithm with DDEs appear rare, with this being possibly the first such example (see S1 Text).

### The system is robust to small evolutionary changes in the magnitude of stochastic effects

**Fig 6:**
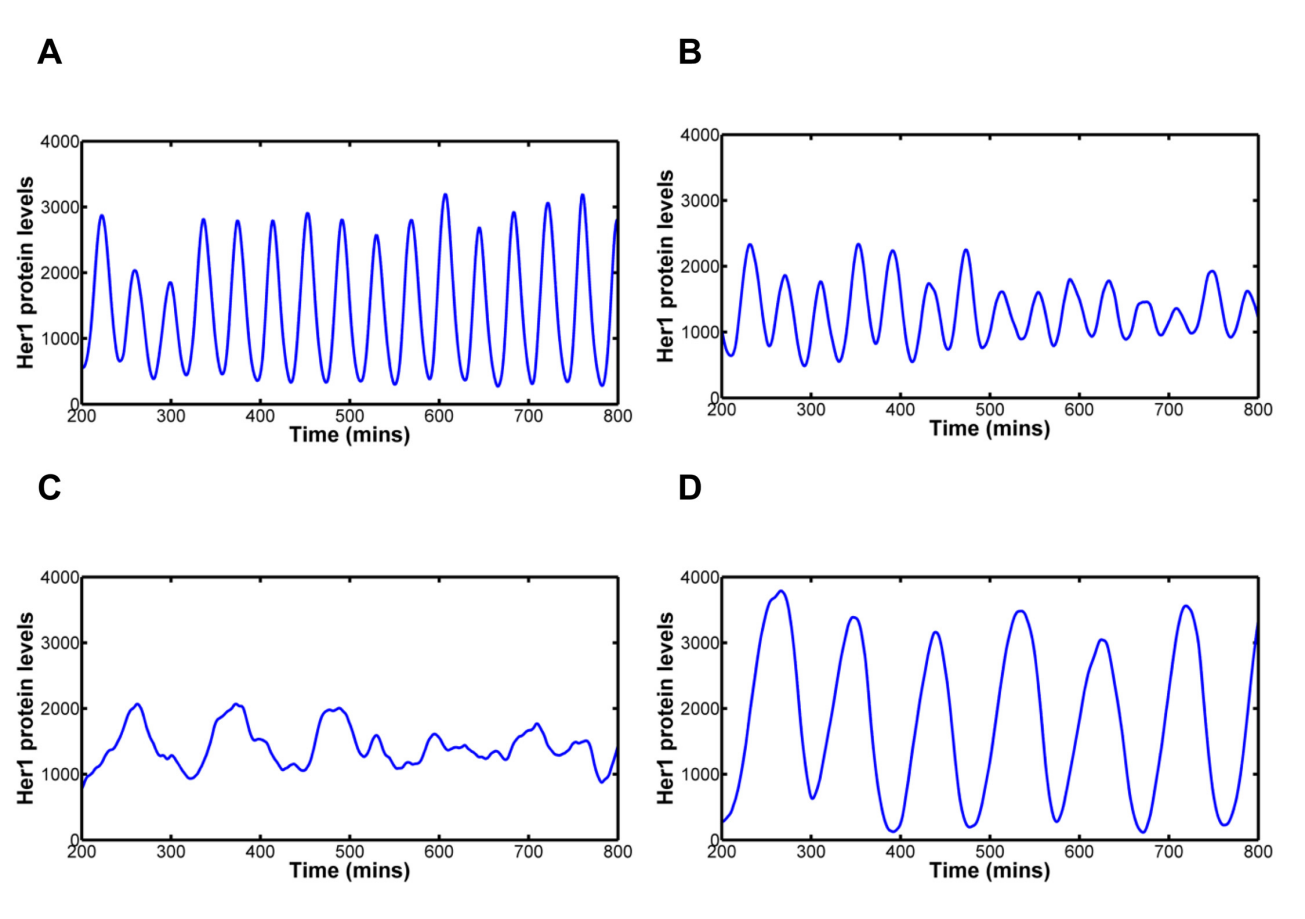
Modelling Notch signalling’s ability to combat increasing levels of stochasticity. In each case we plot the mean Her1 protein levels for 64 cells to illustrate how synchronous the clock oscillations are. Notch signalling is active in each case. 6A: Dissociation parameter, $k_{off_{Her1/7}}=1/6$ min^-1^. Notch signalling can cope with double the magnitude of stochasticity. 6B: Dissociation parameter, $k_{off_{Her1/7}}=1/15$ min^-1^. Notch signalling begins to struggle to keep neighbouring cells oscillating in synchrony. 6C: Dissociation parameter, $k_{off_{Her1/7}}=1/30$ min^-1^. Notch signalling is not strong enough to counteract the levels of stochasticity in *her1/7* gene regulation. Neighbouring cells quickly desynchronise oscillation. 6D: Dissociation parameter, $k_{off_{Her1/7}}=1/30$ min^-1^ while delay (transcription and translation delays and delay in activation of Notch) has been increased threefold. Neighbouring cells once again oscillate in synchrony, but with longer period. As the levels of stochasticity in *her1/7* gene regulation increase, Notch signalling is no longer able to override stochastic effects and keep neighbouring cells oscillating in synchrony. To rescue synchrony, further changes to the system are required, for example, by increasing the magnitude of delay.

Finally, we investigated how robust the inter-cellular Notch synchronisation mechanism is to alterations in the dissociation rate of Her1/7 from their target promoters. This is of interest because *her1/7* are important for many biological processes in addition to the generation of somites and, therefore, their biochemical properties may be subject to diverse evolutionary pressures. Further, it is known that the somite clock has different periods in different organisms. We introduced greater magnitudes of stochastic effects in gene regulation and observed how well the inter-cellular coupling system coped. A modest mutation in the dissociation rates does not make the system fall apart: the system is tolerant to a three or four fold increase in the levels of stochasticity, with neighbouring cells still oscillating in synchrony (Fig 6A and 6B). Increasing the stochastic effects further, causes neighbouring cells to desynchronise as the inter-cellular Notch signalling is not strong enough to override stochastic effects of *her1/7* gene regulation (Fig 6C).

In these limits of increasing stochasticity, other changes in the system are required to rescue synchronous oscillation. The system is able to combat a tenfold increase in stochastic effects and remain in synchrony by increasing the transcription, translation and Notch activation delays threefold (Fig 6D). The period of oscillation is roughly three times longer than that seen in wildtype and hence we would expect the formed somites would also be around three times larger for the same axial elongation rate [76]. Thus, we would expect a reduction in somite number from 31 to around 10, resulting in a number more comparable to frogs.

To conclude, here we present an integrated DDE-Gillespie Algorithm model of oscillating gene expression during somitogenesis. This allows us to determine that the dissociation of the *her1/7* gene repressors is the most likely source of noise in the somite clock that Notch signalling antagonises.

## Methods

### Ethics Statement

Animal experiments were approved by the CRUK London Research Institute Ethical Review Committee (ref. JLE-17/06) and the UK Home Office (Project Licence 80/2081 held by J.L.).

### Fish stocks, mutant and transgenic fish lines

Adult wildtype zebrafish (*Danio rerio*) were kept on a regular light-dark cycle at 27°C. Embryos were maintained at 28.5°C.

### Fluorescent *in situ* Hybridisation and Laser Scanning Microscopy

Zebrafish embryos were paraformaldyde-fixed at 14 hpf and subjected to fluorescent *in situ* hybridisation using a fluorescein- labelled RNA probe against *her1* [77] according to standard protocols. Bound fluorescein-labelled probe was detected using sheep peroxidase-conjugated anti-fluorescein antibody (Perkin Elmer, 1/125) and the TSA Plus Fluorescein system (Perkin Elmer). Specimens were counterstained with DAPI, flat mounted in SlowFade Gold (Invitrogen), and the relevant dorsal to ventral sections of the PSM were imaged with a Zeiss LSM700 confocal microscope at 40x magnification with Nyquist sampling. For the quantification of the *her1* dots representing active transcription of one or both *her1* genes within a cell, stacks of images were generated.

### Quantification of Desynchronisation

The plots of Fig 2 provide the mean Her1 protein levels over 100 cells in addition to the protein levels for five randomly selected cells. If we consider the sample distribution of Her1 protein levels for all 100 cells we can analyse how the distribution changes over time for $k_{off_{Her1/7}}=1/2$ min^-1^ (S5 Movie). The sample distribution oscillates between differing shapes, depending on whether the oscillations are in a trough, peak or somewhere in between the two. In a trough the distribution is heavily localised around low protein levels (S3B) whilst at a peak the distribution is spread over a much larger range of protein levels (S3C). When moving from a peak to trough or vice versa, the shape of distribution is somewhere between the two (S3D and S3E). However, as the oscillations desynchronise, peak and trough distributions become almost identical (S3F and S3G).

To determine when the cells have desynchronised we compare the sample distributions of Her1 protein levels half a period apart (i.e. we compare peak to trough). In the case of our system, this corresponds to a timeframe of approximately 15–16 minutes. We thus compare the sample distribution at a point to the distribution 16 minutes later (e.g. A1 versus A2, B1 versus B2, S3A–S3G Fig). To test for differences in distribution we use a two sample Kolmogorov-Smirnov test. The p-values generated can then be plotted versus time (S3H Fig).

When comparing a peak to a trough (A1 versus A2) the p-value generated is very low, suggesting that the two distributions are different. When comparing the region going from trough to peak (B1) to peak to trough (B2) the results are not statistically significant, as shown by the oscillatory peaks of higher p-values (S3H Fig). However, as the cells desynchronise, the distributions of peak and trough become almost indistinguishable (C1 versus C2) and therefore, the p-value becomes large over all phases of the oscillatory cycle (410 minutes plus, S3H Fig).

We define the cells as being desynchronised and in a salt and pepper pattern after the first instance in which the p-value for no part of the oscillatory cycle drops below 0.1 (S3H Fig, dot dash magenta line). In this instance, the p-value comparing peak and trough must then be greater than 0.1 and the peak distributions have become indistinguishable to the trough distributions. With this methodology, we determine that cells do not desynchronise for $k_{off_{Her1/7}}=1$ min^-1^ and are desynchronised by 406 minutes (13^th^ oscillation) for $k_{off_{Her1/7}}=1/2$ min^-1^, 259 minutes (8^th^ oscillation) for $k_{off_{Her1/7}}=1/3$ min^-1^, 254 minutes (7^th^ oscillation) for $k_{off_{Her1/7}}=1/4$ min^-1^ and 157 minutes (4^th^ oscillation) for $k_{off_{Her1/7}}=1/6$ min^-1^. The last synchronous cycles of oscillation are hence defined to be somewhere between the two oscillations before these salt and pepper regions. For rate and delay constants of Fig 1, cells do not desynchronise for transcription and translation rates, and are desynchronised in 635 minutes for degradation rate, 370 minutes for transcription delay, 451 minutes for transcription delays distributed with the Generalized Pareto distribution, 652 minutes for translation delay and do not desynchronise for number of Hes6 molecules.

### Image Processing of FISH Stacks

The 3D FISH stacks were analysed in MATLAB. Various operations to clean up the image were applied before the nuclei were segmented. S5A Fig gives the nuclei in the middle slice whilst S5B Fig provides a random colour to each segmented nuclei to demonstrate the accuracy of the segmentation. Nuclei within the notochord or outside a manually defined embryo boundary were removed, whilst nuclei in which it was deemed that too great a proportion of the nucleus fell outside the stack boundary were also removed, as seen in S5C and S5D Fig. The dots (*her1* mRNA) were then thresholded within each nucleus such that we could count the total number of dots within each individual nucleus. The nuclei that we analysed alongside the dots signal, expressed in these nuclei, are shown in S5E Fig.

The embryo was divided into 40 intervals, from the posterior of the PSM to the anterior (S5F Fig). By dividing the embryo image into intervals we can thus map the spatial occurrence of nuclei with one *her1* gene expressed and nuclei with two *her1* genes expressed, as a function of distance along the anteroposterior axis. The stripes of gene expression on either side of the notochord occur at an angle to it. The chevrons of the intervals reflect the gradient of these stripes on either side of the notochord. In S5G Fig we have quantified the number of nuclei with one dot (blue signal) and the number of nuclei with two dots (red signal) for each interval in the embryo stack. Smoothing the signals of S5G Fig and transforming the spatial scale from interval number to position along the anterior axis of the PSM results in S5H Fig. The scaling is such that the tail end of the notochord occurs at zero and the anterior end of the PSM occurs at one.

To validate the automatic dot counting, we compared our results to a manual quantification. We manually counted the number of dots for the innermost slice of six of the embryos and compared the results to the number of dots counted in that slice by the automatic three dimensional quantification. In S5I Fig we compare the two for this embryo. Automatic results are in blue (one dot) and red (two dots) and manual results are in cyan (one dot) and magenta (two dots). The broad agreement suggests that our automatic quantification is accurate. A much more in depth description of the image processing procedure can be found in S3 Text.

### Methodology of Quantification of Delay in Expression Between Gene Copies

The length of one formed somite, which can be measured in each embryo, differs slightly from embryo to embryo and is given here by *S*_0_. The time, *T*_0_, to form one somite is determined by the frequency of oscillation of *her1* gene expression in the posterior of the PSM and is approximately 30 minutes for zebrafish at 28°C. Consider a position, *x*, along the anteroposterior axis. The position *x* = 0 corresponds to the tail end of the notochord whilst the position *x* = *L* corresponds to the anterior end of the PSM. Let *v*(*x*) give the speed of forward movement of cells along the anteroposterior axis relative to the tail end of the notochord. We approximated this by linearly interpolating between the known results of *v*(0) = 0 and *v*(*L*) = 1 such that *v*(*x*) = *x* / *L*. Let *S*(*x*) give the local spatial wavelength at position, *x*, measured along the trajectory of cells. Here *x* is the midpoint between one *her1* peak and the next or one *her1* trough and the next. For a spatial delay, *δ x*, centred at position *x* the temporal delay, *δt*, can be calculated such that

$$\delta t=T_{0}\frac{\delta x}{S(x)-v(x)S_{0}}.$$

Distances from different samples can be combined by scaling distances relative to the length of the PSM. Reading the oscillations from anterior to posterior (right to left in Fig 4E and 4F) the increase in frequency of active genes is a reflection of the repressing Her protein dissociating from the gene and hence we quantify the spatial delay, *δ x*, between the wave of cells with one *her1* mRNA molecule and cells with two on this increase. This gives us information specifically regarding the dissociation rate. This delay is best calculated using the inflection point of each wave due to each wave being of different scale and the inflection point being invariant to scaling. The inflection points have been recorded on Fig 4E and 4F and S5H Fig with black circles and the position along the anteroposterior axis, *x*, used for calculation, given by the black cross at the mid-point between the two inflection points. The decrease in frequency of active genes (again reading Fig 4E and 4F from right to left) is a reflection of the repressing Her protein associating to the gene. There is a greater degree of stochasticity here because some genes will be free and just have to associate to Her to become repressed whilst other active genes will be bound to Notch and will first have to dissociate from Notch before associating to Her. All of these events are heavily stochastic.

### Bootstrap Analysis of Sample of Experimental Delay Data

Non-parametric bootstrapping was carried out on the sample of thirteen embryos from which we quantified the delay. We drew samples of size 13, from the original sample, ten thousand times. For each of these redrawn samples, we recorded the mean and standard deviation. Estimates of the population mean (0.1126T_0_) and standard deviation (0.0649T_0_) were found by calculating the means of these statistics, over all samples. The population mean is larger than the sample mean due to the non-symmetric sample distribution. 95% confidence intervals were found by calculating the 5^th^ and 95^th^ percentiles of the sample of means (0.0820 T_0_ ≤ *μ* ≤ 0.1427 T_0_). Due to The Central Limit Theorem, the resulting distributions of population mean and standard deviation are approximately Gaussian.

### The Hybrid Stochastic Delay Deterministic Model

The modelling of Notch signalling incorporates *her1/7* genes, *her1/7* and *delta* mRNA, Her1/7 and Delta proteins and NICD. A hexagonal lattice with periodic boundary conditions is generated such that each cell has six neighbouring cells. Since the signalling is via lateral inhibition, it is these six neighbours that will influence each single cell. *her1/7* gene regulation is modelled stochastically whilst *her1/7* and *delta* mRNA, Her1/7, Delta and Notch proteins are modelled deterministically. The modelling of proteins to *her1/7* is whereby NICD binds as a homodimer to *her*1/7 and Her1 also binds to *her*1/7 as a homodimer. Her7 binds to *her*1/7 as a pair of heterodimers with *hes*6 [50]. Delay in the system is incorporated via DDEs. This delay includes *her1/7* and *delta* transcription delays, Her1/7 and Delta translation delays and the delay in activation of Notch.

A detailed description of the system of reactions and DDEs in addition to how the algorithm is implemented in MATLAB can be found in S1 Text.

Briefly, for this simplified system, the algorithm works as follows:

Step 0) Initialisation. The number of cells in the system is defined and the *her1/7* mRNA, Her1/7 proteins, *delta* mRNA, Delta protein and Notch protein molecule numbers within each cell are initialised either randomly or deterministically. Each cell has two *her1* and two *her7* gene copies. The number of genes bound to Her1/7 or NICD or unbound is initialised either randomly or deterministically. At initialisation time, *t*_0_ = 0. Each cell’s neighbours on the hexagonal lattice are recorded.

Step 1) For the Gillespie Algorithm stage of the modelling, we record all the possible association/dissociation reactions between Her1/7 protein, NICD and *her1/7* genes for all cells in the system. The reactions that can occur are given by Fig 5B. Two random numbers are generated in order to determine the time, and type of the next reaction. The time for the reaction to occur and type of reaction is related, probabilistically, to the population levels of genes and proteins and the reaction rates. For the *i*th Monte Carlo reaction, the time period of reaction is given by *T*_*i*_ and the time the reaction occurs by *t*_*i*_ = *t*_*i*−1_ + *T*_*i*_. Only one single reaction, over all cells will occur in this time interval.

Step 2) The numbers of Her1/7 proteins and *her1/7* mRNA molecules are assumed to be so large, relative to the number of gene copies involved, that many reactions involving solely protein and mRNA will occur between subsequent stochastic gene regulatory reactions. Therefore, we deterministically evolve the system for protein and mRNA levels by solving DDEs for these populations. The *her*1/7 gene levels are fixed over this time interval. The production of *her1/7* is a function of the number of *her1/7* genes switched on. NICD protein levels are determined as a function of neighbouring cells’ Delta levels.

The reaction propensities required to determine the time, *t*_*i*_, and type of the next stochastic reaction are functions of the Her1/7 and NICD populations. Since the population levels of these proteins continuously vary between subsequent stochastic reactions, these reaction propensities also continuously vary. Thus, the time that each reaction takes to occur, *T*_*i*_, and which reaction occurs based on the random numbers generated in Step (1) must be calculated as in the direct method, that incorporates delay, of [75] rather than the standard method of [74]. We thus solve the DDEs over the time period [*t*_*i*−1_, ∞) until we have determined the time, *t*_*i*_, of the next reaction and terminate solution to the DDEs at this time.

Step 3) Having deduced the time period of the next reaction, we determine which reaction occurs using the propensities at the relevant time point as in [75] and update the number of *her1/7* genes bound to Her1/7 and NICD proteins for all cells. We then return to Step (1).

## Supporting Information

S1 Text The mathematical model (DOCX)

S2 Text Parameter Values (DOCX)

S3 Text Extended Description of Image Processing of 3D stacks from FISH Analysis (DOCX)

S4 Text Model Validation: The Gene Regulatory Level (DOCX)

S1 Fig Quantifying the effects of inter-cellular variability in rate constants on cells’ oscillation amplitude and period. Scatter plots of Her1 oscillation amplitude and period versus time, with colour denoting three different cells selected from Fig 1, to demonstrate variability. S1A: Variability in transcription rate. S1B: Variability in translation rate. S1C: Variability in degradation rate. S1D: Variability in transcription delay. This has the largest effect on period of oscillation. S1E: Variability in translation delay. S1F: Variability in cellular numbers of Hes6 molecules. The plots demonstrate that the amplitude and period of each cell’s oscillation smoothly tend to constants, dependent on the magnitude of the rate/delay/Hes6 constant in that single cell. Once the amplitude/period has reached these constants, independent of other cells, the amplitude and period of that cell will remain fixed for all time. Inter-cellular variability in transcription rate and delay dominate variability in amplitude; inter-cellular variability in transcription delay dominates variability in period.
(TIF)

S2 FigQuantifying the effects of stochastic *her1/7* gene regulation on cells’ oscillation amplitude and period.Scatter plots of Her1 oscillation amplitude and period versus time, with colour denoting three different cells randomly selected from Fig 2. S2A: $k_{off_{Her1/7}}=1$ min^-1^. S2B: $k_{off_{Her1/7}}=1/3$ min^-1^. S2C: $k_{off_{Her1/7}}=1/6$ min^-1^. In all three cases the mean amplitude and period for each cell remain fairly constant over time. However, there exist random fluctuations, independent of oscillation stage, around these mean levels. S2D: Plot of standard deviation (20 cells) of phase versus oscillation number for $k_{off_{Her1/7}}=1$ min^-1^ (blue), $k_{off_{Her1/7}}=1/3$ min^-1^ (red) and $k_{off_{Her1/7}}=1/6$ min^-1^ (green). Variability in phase builds up over time. This is slowest for $k_{off_{Her1/7}}=1$ min^-1^ and fastest for $k_{off_{Her1/7}}=1/6$ min^-1^. Random fluctuations in period of oscillation build up over time, increasing variation in phase of oscillation, causing neighbouring cells to desynchronise. This increase in variation is more rapid the greater the level of stochasticity in gene regulation.(TIF)

S3 FigQuantifying the point in time at which neighbouring cells desynchronise.S3A: Mean Her1 protein levels for 100 cells with $k_{off_{Her1/7}}=1/2$ min^-1^ (and blocked Notch signalling) as in Fig 2C. S3B-S3G: Histograms of Her1 protein levels at time points indicated by A1, A2, B1, B2, C1, C2 respectively. When in synchrony, the distribution at low Her1 protein levels (S3B) is noticeably different from the distribution at peak Her1 protein levels (S3C). The difference in distribution when moving from trough to peak Her1 levels (S3D) is not noticeably different to the distribution when moving from peak to trough Her1 protein levels (S3E). As the cells desynchronise, the distributions become indistinguishable (S3F-G). S3H: Plot of p-value versus time for Kolmogorov-Smirnov test comparing distribution of Her1 protein levels to distribution of Her1 protein levels 16 minutes later (approximately half an oscillatory period). For early time, the local minima correspond to p-values comparing trough (e.g. A1) to peak (e.g. A2) and the local maxima to comparing a region between trough and peak (B1) to a region between peak and trough (B2). As the cells desynchronise the p-value becomes large (compare distribution at C1 to at C2). We define the cells to have desynchronised from the first point in time when there is not a significant difference (p-value>0.1) between peak distribution and trough distribution (406 minutes in this case).(TIF)

S4 FigComparison of timeslices of cells’ Her1 protein levels for varying levels of stochastic gene regulation.Using data of Fig 2, we compare low levels of stochasticity (A-C) and high levels of stochasticity (D-F) in gene expression (D-F). Initial conditions are identical in all cells. The time points are selected to represent equivalent points in the oscillatory cycle. A (51 mins) and D (53 mins): peaks of the first oscillations. Cells for both $k_{off_{Her1/7}}$ values are in synchrony. B (132 mins)and E (139 mins): troughs of the third oscillations. $k_{off_{Her1/7}}=1/6$ min^-1^ is desynchronising faster than $k_{off_{Her1/7}}=1$ min^-1^. C (212 mins) and F (220 mins): peak of the sixth oscillations. The cells for $k_{off_{Her1/7}}=1$ min^-1^ are still in synchrony whilst the cells for $k_{off_{Her1/7}}=1/6$ min^-1^ demonstrate a salt and pepper pattern. The cell clocks drift out of synchrony very slowly for $k_{off_{Her1/7}}=1$ min^-1^, the cells do not desynchronise over this time interval and the salt and pepper pattern of *her1* expression is not reached. The cell clocks drift out of synchrony very quickly for $k_{off_{Her1/7}}=1/6$ min^-1^, the cells have desynchronised well before the sixth oscillation and a salt and pepper pattern is apparent. (Compare to the wildtype case of S6A–S6C Fig).(TIF)

S5 Fig Further details of the embryo image processing and delay quantification on a single zebrafish embryo. S5A: Single slice of 3D stack of embryo. S5B: The results of segmentation of nuclei for the 3D stack shown for this single slice. Each segmented nuclei is shown in a random colour. S5C: The notochord and embryo boundaries are manually determined for a number of slices in the 3D stack and the results interpolated throughout. S5D: The resulting slice with nuclei within the notochord, out of the embryo boundary or with too much of the nuclei touching the stack boundary removed. The remaining nuclei are those that we analyse. S5E: The slice with only the nuclei that we analyse, and only *her1* mRNA transcripts that fall within these nuclei included in the image. S5F: The stack is divided into 40 intervals from the posterior of the PSM to the anterior. The gradient of the intervals are based on the gradient of the *her1* mRNA waves. The frequency of nuclei with one dot and two dots is quantified for each interval. S5G: The resulting quantification of frequency of nuclei with one dot and two dots, per interval. S5H: The smoothed signals of S5G Fig. The two dot signal is clearly delayed behind the one dot signal. S5I: Comparison of automatic quantification to manual quantification for the same embryo. A manual count of the number of dots per nuclei in each interval for the innermost single slice of the embryo versus the count per interval in this slice, derived from the three dimensional quantification. The signals are similar, suggesting that the automatic quantification is accurate.
(TIF)

S6 FigComparison of timeslices of Her1 protein levels for active Notch signalling and deactivated Notch signalling.Data is from Figs 2 and 5. Activated Notch signalling (A-C) is compared to the case of deactivated Notch signalling (D-F). The dissociation rate is set as $k_{off_{Her1/7}}=1/3$ min^-1^. A (216 mins) and D (52 mins): peaks of oscillation one. In the case of active Notch signalling, oscillation one has been arbitrarily defined as an oscillation after Notch signalling has overridden random initial conditions. The cells oscillate in synchrony in both cases. B (388 mins) and E (200 mins): troughs of oscillation 5. The cells for active Notch oscillate in synchrony whilst the cells for deactivated Notch are drifting out of synchrony. C (518 mins) and F (325 mins): The cells for active Notch oscillate in synchrony whilst those for deactivated Notch have desynchronised and a salt and pepper pattern is apparent. Notch signalling keeps neighbouring cells oscillating in synchrony whilst, in its absence a salt and pepper pattern gradually emerges.(TIF)

S7 Fig Quantification of delay in expression between *her1* gene copies in a cell from simulated data. S7A–S7D Fig correspond to active Notch signalling. S7A: Number of active *her1* genes versus time, for a single cell. S7B: Plot of frequency of cells with one *her1* gene copy expressed (blue signal) and two *her1* gene copies expressed (red signal) for the output of the mathematical model (as was done for the experimental data). S7C: Smoothed version of S7B. S7B and S7C Fig demonstrate that there is a delay between the expression of the first *her1* gene copy and the second in a cell. The black circles in S7C Fig demonstrate the inflection points used to calculate the delay. S7D: Box and whisker plots comparing sample delays of experimental data (left) to simulated data with stochastic gene regulation (centre) and simulated data with stochastic gene regulation and inter-cellular variability (right). The inter-cellular variability is introduced in the transcription, translation and degradation rates and number of Hes6 molecules at the magnitudes described in Fig 1. The whiskers give the maximum and minimum, the box the lower quartile and upper quartile, the solid red line the median and the red dot-dash line the mean. The left axis gives the scale in terms of proportion of time to form one somite, the right in terms of minutes, on the assumption it takes 30 minutes to form one somite. The boxplots demonstrate that the average delay is well recreated by the mathematical model incorporating stochastic gene regulation, with or in the absence of inter-cellular variability, but the probability distribution is not. The lack of impact when adding inter-cellular variability suggests that delay in gene expression is a consequence of stochasticity in repressor/DNA dissociation and that the quantification of the stochastic dissociation rate is robust to inter-cellular variability. S7E: Plots of mean Her1 protein levels for 100 cells for three different embryos when Notch signalling is deactivated for the case of stochastic gene regulation and inter-cellular variability as in S7D Fig. The cells desynchronise in 6–8 oscillations similarly to Fig 2D and 2G demonstrating that the addition of inter-cellular variability does not drastically alter the desynchronisation rates imposed by stochastic gene regulation. S7F: The frequency of cells with one *her1* gene expressed (blue line) and two *her1* genes expressed (red line) versus time, when Notch signalling is deactivated. The signals descend to noise from oscillatory behaviour that occurs when Notch signalling is active. There is good agreement between the mathematical model and experimental data in terms of delay in expression between the first *her1* gene copy in a cell and the second. This demonstrates that the gene regulatory level in the model is behaving realistically and further validates both the plausibility of the mathematical analysis and the argument that delay is a consequence of stochasticity in gene regulation.
(TIF)

S1 Table Statistics of the samples of delay from both the experimental data and the simulated data with and without inter-cellular variability. Expressed in terms of proportion of time to form one somite.
(DOCX)

S2 Table Raw delay data for both the experimental data and the simulated data with and without inter-cellular variability. Expressed in terms of proportion of time to form one somite.
(DOCX)

S1 MovieDeactivated Notch signalling, 100 cells, $k_{off_{Her1/7}}=1$ min^-1^.Oscillating Her1 protein levels for cells displayed on a hexagonal lattice. Dark green corresponds to low Her1 protein levels and light green to high Her1 protein levels. The amplitudes have been normalised to the maximum and minimum of each cell’s amplitude. Initial conditions are identical for all cells. The stochasticty in gene regulation is low and so the desynchronisation occurs very slowly. The salt and pepper pattern does not occur over this time period.(AVI)

S2 MovieDeactivated Notch signalling, 100 cells, $k_{off_{Her1/7}}=1/6$ min^-1^.Oscillating Her1 protein levels for cells displayed on a hexagonal lattice. Dark green corresponds to low Her1 protein levels and light green to high Her1 protein levels. The amplitudes have been normalised to the maximum and minimum of each cell’s amplitude. Initial conditions are identical for all cells. The stochasticity in gene regulation is high resulting in the cells desynchronising quickly and the salt and pepper pattern being reached after 160 minutes.(AVI)

S3 MovieDeactivated Notch signalling, 100 cells, $k_{off_{Her1/7}}=1/3$ min^-1^.Oscillating Her1 protein levels for cells displayed on a hexagonal lattice. Dark green corresponds to low Her1 protein levels and light green to high Her1 protein levels. The amplitudes have been normalised to the maximum and minimum of each cell’s amplitude. Initial conditions are identical for all cells. The cells gradually desynchronise and the salt and pepper pattern is reached after 260 minutes.(AVI)

S4 MovieActive Notch signalling, 64 cells, $k_{off_{Her1/7}}=1/3$ min^-1^.Oscillating Her1 protein levels for cells displayed on a hexagonal lattice. Dark green corresponds to low Her1 protein levels and light green to high Her1 protein levels. The amplitudes have been normalised to the maximum and minimum of each cell’s amplitude. Initial conditions are random for all cells. Notch signalling is strong enough to override random initial conditions and force neighbouring cell clocks into synchrony. From then on (200 minutes plus) cells oscillate in synchrony throughout.(AVI)

S5 MovieTime evolving frequency distribution of Her1 protein levels for $k_{off_{Her1/7}}=1/2$ min^-1^.The distribution oscillates between low Her1 protein levels with low variability and high Her1 protein levels with high variability. As neighbouring cells desynchronise, the oscillatory behaviour of the frequency distribution is destroyed. See Figs 2C and S3.(AVI)
