# Supplementary material for: Stochastic Regulation of her1/7 Gene Expression Is the Source of Noise in the Zebrafish Somite Clock Counteracted by Notch Signalling
Source: PLoS Comput Biol. 2015 Nov 20;11(11):e1004459. doi: 10.1371/journal.pcbi.1004459 (PMC4654481; doi:10.1371/journal.pcbi.1004459)
Supplement: S1 Text — (DOCX) [file pcbi.1004459.s001.docx]

**S1 Text. The mathematical model**

**Hybridisation of DDEs with The Gillespie Algorithm**

We have developed a mathematical model that incorporates *her1/7* genes, mRNA and proteins, *delta* mRNA and proteins and NICD. Levels of mRNA and protein in the system are large such that we can model these aspects deterministically using differential equations. Delay in transcription and translation play a prominent role in the generation of oscillation and are incorporated via DDEs. Within each cell there are two *her1* genes and two *her7* genes. The low numbers of genes in each cell mean that we cannot ignore stochastic effects and we hence model reactions involving these genes using a modified Gillespie Algorithm [[74](#_ENREF_74), [75](#_ENREF_75)].

The first algorithms that modelled chemical reactions (without any form of delay) stochastically were developed such that the next reaction to occur was determined randomly. However, the time period for this reaction to occur over was fixed [[78](#_ENREF_78)]. Therefore, in the words of Gillespie [[74](#_ENREF_74)] when introducing The Gillespie Algorithm, such a method ‘becomes exact in the limit of the timestep tending to zero, but unfortunately the efficiency of the procedure becomes nil in that same limit.’ A more rigorous approach, The Gillespie Algorithm simulates chemical reactions in such a way that both the next reaction to occur and the time taken for that reaction to occur are both determined randomly. Attempts have been made to increase the speed of The Gillespie Algorithm, when reactions occur on vastly different timescales, by partitioning the reactions into fast and slow reactions [[79-83](#_ENREF_79)]. This would be the case when considering reactions involving large numbers of mRNA and protein molecules, which would occur at a much higher frequency and could be modelled deterministically, alongside those involving small numbers of genes, which should be modelled stochastically.

As described in the Discussion, previous hybrid models that incorporated stochastic gene regulation with deterministic DDEs did so in such a way that each stochastic reaction will occur over a fixed timestep [[20](#_ENREF_20), [50](#_ENREF_50)]. Such methods have produced insightful analysis but, they suffer from the same criticisms of fixed timesteps, described by Gillespie, above. The population levels of active genes can change only at each fixed timestep which will average out some of the stochastic effects and result in behaviour intermediate between deterministic and stochastic models. Our investigation focused on the effects of stochasticity in the system and we therefore desired to model it as rigorously as possible. Incorporating a modified Gillespie Algorithm, for the modelling of stochastic gene regulation, results in both the next reaction to occur and the time that this reaction takes to occur being made random, resulting in the stochastic her1/7 gene regulation in our hybrid model being truly random. In the past, The Gillespie Algorithm and chemical master equation have both been adapted to incorporate delay [[63](#_ENREF_63), [65](#_ENREF_65), [66](#_ENREF_66), [75](#_ENREF_75)]. However, this hybridisation of the Gillespie Algorithm with DDEs appears (to the best of the authors’ knowledge) to be the first such example.

**A Description of the Resulting Model**

The reaction schemes modelled and delay differential equations are introduced as in [[50](#_ENREF_50)]. The binding of Her1/7 proteins to *her1/7* inhibits expression of *her1/7*. In our model, Her1 binds to *her*1/7 as a homodimer. Her7 binds with *her*1/7 as a pair of heterodimers with *hes*6. NICD homodimers compete with Her1/7 proteins to bind to *her1/7*. When NICD is bound to *her1/7* then Her1/7 dimers cannot bind and expression of *her1/7* occurs (as is the case when *her1/7* is free). The reactions considered are given by

Here, $G_{r}$refers to *her1/7* genes in their unbound state,$HG_{r}$to the genes when Her proteins are bound to them (repressing their activity), $N$to NICD and $NG_{r}$to NICD bound to the genes (in this state the genes are still active). There is a slight abuse of notation in that the Her1 and Her7-Hes6 binding reactions should be noted separately. In this case $H$refers to either the Her1 homodimer or the Her7-Hes6 heterodimer. The association parameters, $k_{on},$are derived as a ratio of the dissociation rates to the relevant critical protein concentrations for inhibition of genes.

These reactions are modelled stochastically using The Gillespie Algorithm, modified to incorporate the additions of [[75](#_ENREF_75)], to reflect time varying reaction propensities of equations 1-8, due to the dynamic evolution of the $H$ and $N$protein populations. The Gillespie Algorithm is constructed from Markov Chain theory. At each Monte Carlo timestep a random number is generated that determines the point in time that the next reaction occurs. This timescale is proportional to the number of molecules in the system and is derived from the exponential distribution. A second random number is then generated to determine which reaction occurs next. Reactions with larger reaction rates or those involving greater numbers of molecules are more likely to occur.

The DDEs for *her1/7* mRNA, Her1/7 protein, *delta* mRNA, Delta protein and NICD protein are given by the equations

The subscript, $j,$records the cell index. The total number of gene copies switched on in a cell is given by $g,$ $m$gives the mRNA levels and $p,$the protein levels. The additional subscripts for each variable reflect whether it is a *her1/7*, *delta* or Notch element. The α terms refer to mRNA synthesis rate, λ to mRNA degradation rate, β to protein synthesis rate and η to protein degradation rate. Transcription delay is given by $\tau_{m}$and translation delay by $\tau_{p}.$Equation (13) gives the production of *delta* mRNA with production being a Hill function of Her1/7 and Hes6 protein (effectively assumed to be constant in this case). The values $pcrit\delta_{h1},$ $pcrit\delta_{h7}$ and $pcrit\delta_{h6}$correspond to the critical protein concentrations for inhibition of the *delta* gene whilst $hillh1,$ $hillh7$and $hillh6$provide the stoichiometry of the proteins binding to DNA. Equation (15) gives the differential equation for NICD production. The production is as a function of the amount of Delta protein in neighbouring cells. The summation is over the Delta protein level, in all neighbouring cells, $\tau_{p_{N}}=20$minutes previously. In most cases, $N_{\max}=N_{6}$ due to our lattice being hexagonal, the exception being the cells on the boundary if we do not apply periodic boundary conditions. The critical protein concentration for inhibition of Notch by Delta is given by $pcritN_{\delta}.$

Our hybrid model of DDEs and The Gillespie Algorithm has been developed using MATLAB. We simulate the Gillespie Algorithm over a number of Monte Carlo steps. The time in the system at the $i-1$th Monte Carlo timestep is given by $t_{i-1}.$At the $i$th Monte Carlo step, we randomly determine how long, $T_{i},$the next stochastic reaction occurs over (evolving time to $t_{i}=t_{i-1}+T_{i}$) and which reaction it is that occurs next. The DDEs are solved over the time interval $\left[t_{i-1},\text{ }t_{i}\right]$using the history of past gene, mRNA and protein levels. Due to the protein levels continuously varying over this interval and the fact that the stochastic reactions in equations 1-8 incorporate these dynamically varying variables it is possible to exactly determine the time of the next reaction, $t_{i},$and which reaction occurs only whilst the DDEs are in the process of being solved.

Our algorithm works as follows:

Step 0) Initialisation. An m by n hexagonal lattice of cells is generated and each cell’s neighbours recorded. We use periodic boundary conditions. Initial population levels of mRNA and protein in each cell are set. This can be either random or uniform, such that all values are equal in every cell. The number of *her1/7* genes unbound or bound to NICD or Her1/7 in each cell is set. Again, this can be random or uniform over all cells.

Step 1) Using MATLAB’s inbuilt dde23 solver, we solve the system deterministically up to the period of time of maximum delay. Maximum delay in our current model is $\tau=20$minutes when Notch signalling is active (seven minutes when it is not). This solution provides us with a history function required during the initial, stabilisation period of the model. Over this period of twenty minutes, the *her1/7* genes remain in whichever bound state they are at initialisation. The requirement for this time period in deterministic initialisation and the initial conditions results in the system taking time to settle into its true structural behaviour. Our Gillespie based stochastic reactions will then occur at times $t_{0},t_{1},t_{2},t_{3},\ldots.$ To keep track of the time of *her1/7* gene based reactions, we set $t_{0}=0$ and $t_{1}=20$ to take account of the history function derived in this step. The time between reactions $i$ and$i-1$ is then given by $T_{i}=t_{i}-t_{i-1}.$ The time step, $T_{1},$ is given by the maximum delay over the deterministic solving period and hence our first Monte Carlo step occurs at $i=2$ where the time period of the reaction is given by $T_{2},$resulting in $t_{2}=20+\text{ }T_{2}.$

Step 2) We generate random numbers, $r_{2},$ to determine the time of reaction, $t_{2},$ and $r_{1},$ to determine which reaction between *her1/7* genes, Her1/7 dimers and NICD occurs next from reactions (1) to (8). Only one cell, $cell_{j},$ undergoes a reaction at each Monte Carlo step. The calculation of this time point, $t_{2},$ and type of reaction is found using the methods of [[75](#_ENREF_75)] and thus requires us to solve the DDEs beyond the timepoint $t_{1}$before we have determined $t_{2}.$

Step 3) We solve the deterministic DDEs for *her1/7* mRNA, Her1/7 protein, *delta* mRNA, Delta protein and NICD for all cells over the time interval $[t_{1},\infty)$ until we have determined the time, $t_{2}=t_{1}+\text{ }T_{2},$ of the next reaction using the methods of [[75](#_ENREF_75)]. Over this time interval, *her1/7* gene states are fixed at $t_{1}$ levels. We terminate solution to the DDEs at this derived time point, $t_{2}.$ It is the determination of this time point where Cai’s direct method that incorporates delay [[75](#_ENREF_75)] differs most from the original Gillespie Algorithm [[74](#_ENREF_74)]. Determining the next reaction to occur is equivalent to in the standard Gillespie Algorithm, however with reaction propensities adjusted according to [[75](#_ENREF_75)]. Solving these DDEs in MATLAB is not trivial. MATLAB’s inbuilt dde23 solver is insufficient for the task at hand, so we instead use MATLAB’s ode23 solver. For the functional reliance of the differential equations on population levels at an earlier point in time (the delay), we record history vectors, whereby we shift the gene, mRNA and protein population vectors along by an amount corresponding to their respective delays. See below for further details.

Step 4) In $cell_{j},$the gene population levels are kept fixed over the time period $\left[t_{1},t_{2}\right).$ Only at time $t_{2}=t_{1}+\text{ }T_{2}$ do we update the *her1/7* gene populations in $cell_{j}$ according to which of (1) to (8) reactions occurred. The gene populations in all other cells do not change over this time period. Following this update, we return to Step 2 to determine the time period and type of reaction for $i=3$ solving the DDEs over $[t_{2},t_{3}]$before updating the gene populations accordingly. The algorithm then continues until a desired period of time or number of Monte Carlo steps is reached.

**Technique of Implementing the Hybrid Algorithm in MATLAB**

MATLAB’s inbuilt dde23 solver is insufficient to solve our systems of DDEs for two reasons. Firstly, the *her1/7* gene expression numbers in each cell are discontinuous, varying between 0,1 and 2 and changing instantaneously at each Gillespie timestep. Secondly, the time interval we solve over at each step is random and consequently varies greatly in size. Therefore, we transform our DDEs into ordinary differential equations (ODEs). Instead of incorporating the delay functions in the equations explicitly, we include input history vectors into a system of ODEs. To derive these input history vectors we shift our gene, mRNA and protein vectors along by an amount corresponding to the delay in transcription and translation of each component. For example, Her1 protein production is a function of the *her1* mRNA levels 1.1 minutes earlier. We translate the *her1* population levels along by 1.1 minutes and then use this as the input history. For each timestep that ode23 solves over, we interpolate the input history vector to derive the correct scalar input at that point in time. ode23 is very fast in solving our differential equations as it has a lower accuracy than other solvers. It is able to deal with moderately stiff problems of which ours is, due to the discontinuous shifts in gene expression. Due to the discontinuities in the gene expression vectors, to solve our system of equations over the time interval $\left[t_{i},t_{i+1}\right]$ we must break this interval up into much smaller intervals between these discontinuities. For the period of time, $\left[t_{i},t_{i+1}\right]$ that we solve over, we record all the points in time that there are discontinuous changes in *her1/7* gene expression numbers from the history vectors of gene populations. We then divide the period $\left[t_{i},t_{i+1}\right]$ into intervals according to where these discontinuities occur. We solve the ODEs over each of these subintervals, using the deterministic solution to the previous subinterval as the initial conditions for the next subinterval. The *her1/7* gene expression levels are thus constant over each subinterval. In this way we are able to sidestep the problems of both the discontinuities in the history function for *her1/7* gene expression and the largely variable time interval $\left[t_{i},t_{i+1}\right].$ To speed up MATLAB’s interpolation over these input history vectors, only the portion of the vectors that cover the given subinterval are input into the ODE solver. In addition, when the difference in time between the current timepoint and the oldest timepoint in the solution reaches a level greater than the maximum delay (20 minutes when Notch is active), the data is saved. All data in the solution vectors, relating to the period of time older than the maximum delay required by our solvers, is then removed. This avoids the solution vectors becoming too large and consequently vastly slowing down the solver.

**Simplified Deterministic System of Her1/7 Oscillations in the Absence of Stochastic Gene Regulation**

The model required, when not considering stochastic gene regulation, is vastly simplified. This is the system required to consider the effects of inter-cellular variability in reaction rate constants and delay constants in the *her1/7* feedback loop. The model incorporates only DDEs and these model just *her1/7* mRNA and Her1/7 protein. The system of equations are given by

whereby variable and parameter descriptions are equivalent to those found above. The only exception being $\alpha_{h1/7}$which we set equal to 33 since the production rate should reflect the fact that there are two genes in each cell (this is implicit in the stochastic-deterministic model above). The production rates of *her1/7* mRNA side-track modelling gene regulation and rely on protein concentrations at prior points in time.
