# Supplementary material for: Stochastic Regulation of her1/7 Gene Expression Is the Source of Noise in the Zebrafish Somite Clock Counteracted by Notch Signalling
Source: PLoS Comput Biol. 2015 Nov 20;11(11):e1004459. doi: 10.1371/journal.pcbi.1004459 (PMC4654481; doi:10.1371/journal.pcbi.1004459)
Supplement: S2 Text — (DOCX) [file pcbi.1004459.s002.docx]

**S2 Text. Parameter Values**

Parameter values are generally based on those found in [[18](#_ENREF_18), [20](#_ENREF_20), [49](#_ENREF_49), [50](#_ENREF_50)].

Critical protein concentrations for inhibition of genes (in molecules per cell):

$pcrith1_{h1/7}=100$ (Critical concentration of Her1 for binding to *her1/7* regulatory locus),

$pcrith7_{h1/7}=100$ (Critical concentration of Her7 for binding to *her1/7* regulatory locus),

$pcrith6_{h1/7}=100$ (Critical concentration of Hes6 for binding to *her1/7* regulatory locus),

$pcrit\delta_{h1}=100$ (Critical concentration of Her1 for inhibition of *delta* gene expression),

$pcrit\delta_{h7}=100$ (Critical concentration of Her7 for inhibition of *delta* gene expression),

$pcrit\delta_{h6}=100$ (Critical concentration of *hes6* for inhibition of *delta* gene expression),

$pcritN_{\delta}=10000$ (Critical concentration of Delta for activation of Notch. A large value represents the condition that Delta levels are far below the saturating level for Notch activation),

$pcrith_{N}=50$ (critical concentration of NICD protein for binding to *her1/7* regulatory locus in molecules per cell).

mRNA synthesis rates:

$\alpha_{h1}=16.5$min^-1^ (Maximal synthesis rate of *her1*),

$\alpha_{h7}=16.5$min^-1^ (Maximal synthesis rate of *her7*),

$\alpha_{\delta}=33$min^-1^ (Maximal synthesis rate of *delta*).

Protein synthesis rates:

$\beta_{h1}=9.2$min^-1^ (Rate of synthesis of Her1 per molecule of *her1* mRNA),

$\beta_{h7}=9.2$min^-1^ (Rate of synthesis of Her7 per molecule of *her7* mRNA),

$\beta_{\delta}=9.2$min^-1^ (Rate of synthesis of Delta per molecule of *delta* mRNA),

$\beta_{N}=0.1\times pcritN_{\delta}=1000$min^-1^ ($\beta_{N}/pcritN_{\delta}$is the rate of synthesis of NICD per molecule of Delta when Delta is well below its critical value).

Parameters for delays, lifetimes and dissociation rates (with the exception of our value for $k_{off_{Her1/7}})$are those found in [[49](#_ENREF_49), [50](#_ENREF_50)] which are loosely based on [[18](#_ENREF_18), [20](#_ENREF_20), [48](#_ENREF_48)].

mRNA degradation rates (inverse of lifetime):

$\lambda_{h1}=0.23$min^-1^ (Degradation rate of *her1* mRNA),

$\lambda_{h7}=0.23$min^-1^ (Degradation rate of *her7* mRNA),

$\lambda_{\delta}=0.23$min^-1^ (Degradation rate of *delta* mRNA).

Protein degradation rates (inverse of lifetime):

$\eta_{h1}=0.23$min^-1^ (Degradation rate of Her1 protein),

$\eta_{h7}=0.23$min^-1^ (Degradation rate of Her7 protein),

$\eta_{\delta}=0.23$min^-1^ (Degradation rate of Delta protein),

$\eta_{N}=0.23$min^-1^ (Degradation rate of Notch protein).

Transcription delays:

$\tau_{m_{h1}}=7$ minutes (Delay in transcription of *her1*),

$\tau_{m_{h7}}=7$minutes (Delay in transcription of *her7*),

$\tau_{m_{\delta}}=7$minutes (Delay in transcription of *delta*).

Translation delays:

$\tau_{p_{h1}}=1.1$minutes (Delay in translation of *her1*. Data for ovalbumin synthesis in chick [[78](#_ENREF_78)]),

$\tau_{p_{h7}}=0.7$minutes (Delay in translation of *her7*. Data for ovalbumin synthesis in chick [[78](#_ENREF_78)]),

$\tau_{p_{\delta}}=20$minutes (Delay in translation of *delta*),

$\tau_{p_{N}}=2$minutes (Delay in activation of Notch).

Stoichiometric coefficients for binding to DNA:

$hillh1=2$ (Her1 binding to DNA),

$hillh7=2$ (Her7 binding to DNA),

$hillh6=2$ (*hes6* binding to DNA).

Concentration of additional proteins:

$p_{h6}=100$ (number of Hes6 protein molecules, assumed constant, set at same value as in [[49](#_ENREF_49), [50](#_ENREF_50)] ).

Dissociation rates:

$k_{off_{Her1/7}}=1/3$min^-1^ (Rate constant for dissociation of Her protein from the *her1/7* promoter/enhancer. Taken from our image analysis of Fig. 4. Elsewhere in the paper we vary this parameter),

$k_{off_{N1}}=1$min^-1^ (Rate constant for dissociation of NICD protein from *her1* promoter/enhancer).

$k_{off_{N7}}=1$min^-1^ (Rate constant for dissociation of NICD protein from *her7* promoter/enhancer).

Association rates:

Values for $k_{on}$ are as in [[50](#_ENREF_50)][[34](#_ENREF_34),[50](#_ENREF_50)].

Notch binds to *her1/7* as a homodimer, resulting in the quadratic term:

$$k_{on_{N1/7}}=\frac{k_{off_{N1/7}}}{pcrith_{N}^{2}}.$$

The $k_{on}$ rate of Her1/7 to their inhibitory sites on *her1/7* DNA are made up of two parts:

The part for Her1 to bind to *her1/7* as a homodimer

$$k_{on_{Her1}}=\frac{k_{off_{Her1/7}}}{pcrith1_{h1/7}^{2}},$$

and the part for Her7 to bind as a pair of heterodimers with *hes6*

$$k_{on_{Her7}}=\frac{k_{off_{Her1/7}}}{pcrith7_{h1/7}^{2}\times pcrith6_{h1/7}}.$$
