# Supplementary material for: Stochastic Regulation of her1/7 Gene Expression Is the Source of Noise in the Zebrafish Somite Clock Counteracted by Notch Signalling
Source: PLoS Comput Biol. 2015 Nov 20;11(11):e1004459. doi: 10.1371/journal.pcbi.1004459 (PMC4654481; doi:10.1371/journal.pcbi.1004459)
Supplement: S3 Text — (DOCX) [file pcbi.1004459.s003.docx]

**S3 Text. Extended Description of Image Processing of 3D stacks from FISH Analysis**

The image analysis was carried out in MATLAB on 8-bit images. Initially, a histogram equalisation of intensity was carried out on both the nuclear channel and dots channel to reflect the fact that the intensity of pixels decreases with depth into the stack. In the case of the dots channel, the maximum intensity was found for each slice. The dots channel was then saturated at the minimum value of these maximums to stop a small number of outlier pixel intensities having a large effect on the histogram equalisation of the entire stack. Both the nuclear channel and dots channel were then smoothed with Gaussian functions with standard deviations equal to the mean radii of nuclei and dots respectively. Watershedding was applied to the nuclear channel. Basins which fell below a certain volume threshold or in which the maximum intensity of pixels was below a certain threshold were removed. We then locally thresholded, within each basin of attraction, to segment each nucleus in the stack. Several different thresholding methods were tried and Otsu’s method was deemed the most accurate after comparison to human observation.

The boundaries of the embryo and notochord were manually recorded, using MATLAB’s improfile setting, for ten evenly spaced slices within each stack and interpolation carried out between them. Nuclei whose centroids fell outside our embryo boundary or inside the notochord boundary were removed from the analysis. Nuclei that touched the boundary of the stack were removed if they fulfilled certain criteria. Removing every nucleus that touched a boundary led to too few nuclei being analysed per embryo and caused the signal to noise ratio to become very low. Hence, it was decided that we would keep nuclei in which it was estimated that they had approximately 95% of their cell volume within the image. This threshold enabled us to keep the majority of our nuclei and also maintain a high dot counting error tolerance within each nucleus. To estimate whether a nucleus on the image boundary was missing at most 5% of its volume we made the assumption that each nucleus was approximately spherical. For a pixelated spherical object of radius 10 pixels (the approximate radius of a nucleus in our analysis), a missing volume of 5% corresponds to approximately 4% of the total volume of the recorded sphere touching the image boundary. Therefore, we included a threshold in which we excluded nuclei if more than 4% of the recorded nucleus was on the stack boundary.

The embryo was then divided into forty intervals along the anteroposterior axis of the PSM. To determine the gradient of the intervals, we selected ten evenly spaced slices from the image. For each slice, the gradient of the intervals was determined by the gradient of the mRNA molecules on both the left and right of the notochord, independently of one another. Interpolation of the intervals was then carried out over the whole stack between the ten slices. Each nucleus was defined to be in whichever interval its centroid was located in.

We then considered the dots channel locally within each nucleus object by multiplying the dots channel by a binarised mask of the nuclear channel. Watershedding was then carried out on the resulting image of dots. Shallow intensity basins, in which the difference between the maximum and minimum intensity was less than a threshold value were removed. Small objects and/or those with maximum intensity below a threshold value were removed as suspected noise in the image acquisition and analysis. The remaining watershedded basins were taken to be the mRNA dots within each nucleus.

From this data the number of nuclei with 0, 1, 2 or more dots in each interval could be determined and the spatial periodic data recovered. A small number of cells appeared to contain three or four dots and it is presumed that these cells were in G2 phase of the cell cycle. A smaller number still, appeared to contain more than four dots, which is most likely a consequence of noise in the image acquisition or image analysis. Cells that contained such events were ignored since it was too costly in terms of time to investigate each occurrence and the overall impact on one dot and two dot signals was minimal.

Once the one and two dot signals were acquired for each embryo, the signals were smoothed using a moving average filter before the analysis to derive the temporal information was carried out. The frequency data over forty intervals was interpolated over a much finer grid. The interpolated data was then split into two signals, one for each peak. Each of the resulting signals was then smoothed separately (to avoid interference from the other peak), using MATLAB’s filter function with a window-size appropriate to the signal. The two smoothed peaks were then recombined and the interpolated interval vector transformed to be measured from zero (tailend of notochord) to one (anterior end of the PSM).
