# Supplementary material for: Stochastic Regulation of her1/7 Gene Expression Is the Source of Noise in the Zebrafish Somite Clock Counteracted by Notch Signalling
Source: PLoS Comput Biol. 2015 Nov 20;11(11):e1004459. doi: 10.1371/journal.pcbi.1004459 (PMC4654481; doi:10.1371/journal.pcbi.1004459)
Supplement: S4 Text — (DOCX) [file pcbi.1004459.s004.docx]

**S4 Text. Model Validation: The Gene Regulatory Level**

We considered the number of cells with one or two *her1/7* gene copies expressed versus time in the simulated data, in comparison to the experimental data of Fig. 4. If the stochastic gene regulation within our model is working as we have so far described then, when Notch signalling is active, the delay in expression of approximately three minutes between the first gene copy being expressed in a cell and the second should be recapitulated. This would provide further confidence that our system desynchronises at the correct rate directly because of the way in which the *her1/7* genes are regulated stochastically in the system. S7A Fig. shows how the number of active *her1* genes in a single cell fluctuates over time. S7B Fig. and S7C Fig. give the raw and smoothed signals for number of cells with one *her1* gene copy expressed (blue line) and the number of cells with two *her1* gene copies expressed (red line). It is clear that the two dot signal is delayed behind the one dot signal.

Data on the calculated delays from this simulated data can be found in S1 and S2 Tables. For the period of time in which Notch has overridden the effects of the random initial conditions we observed a mean delay of 3.5 minutes (or 0.12 as a proportion of the time to make one somite). This corresponds well with an estimated population mean delay of 3.4 minutes from the experimental results (or 0.11 as a proportion of time to make one somite). In S7D Fig. we compared the box and whisker plots of the experimental data to the simulated data. Carrying out the Mann-Whitney U test between the two samples provides a p-value of 0.9443. We cannot reject the hypothesis that the two populations are the same. This is what we would expect, if the stochastic gene regulation within our model was setup correctly. However, the model has been less successful in recreating the overall probability distribution of the delay. This increased experimentally observed variation is likely, in part, to be a consequence of variation in experimental measurement. However, other random biological factors, such as the stochastic regulatory nature of recruitment of further transcription factors, inter-cellular variability in transcription, translation, degradation and delay parameters, the inclusion of stochastic gene regulation of *delta* and noise resulting from cell division could all increase the variability in this delay. As such, we introduced inter-cellular variability into this model to investigate its effect on distribution of delay. We introduced inter-cellular variability in transcription, translation and degradation rates and number of Hes6 molecules at the same magnitudes as described in Fig. 1. The mean delay of 3.2 minutes (0.11 as a proportion of time to make one somite) and p-value of 0.6447, corresponding to a Mann-Whitney U test with the experimental data, again suggest little difference between the experimental data and simulated data. However, the corresponding box and whisker plot (S7D Fig.) again shows that the variability in delay has not been well approximated by this model suggesting that the increased experimental variation is not a consequence of the inter-cellular variability that we have introduced. In S7E Fig. we consider the case of added inter-cellular variability to the stochastic gene regulatory model when Notch signalling has been deactivated. The results demonstrate that cells still desynchronise in 6-8 oscillations, the same magnitude observed in the absence of inter-cellular variability. S7D-E Figs add weight to the arguments that stochasticity in the repressor/DNA dissociation is the driving factor behind delay in *her1* gene expression and illustrate that inter-cellular variability does not significantly alter the desynchronisation rate due to this stochasticity. Therefore, the quantification of the dissociation parameter, $k_{off_{Her1/7}},$should be robust to this inter-cellular variability.

Finally, returning to the case of no inter-cellular variability, when Notch signalling is blocked, S7F Fig. demonstrates that the frequency of cells with one (blue) or two (red) genes active descends to noise from their oscillatory behaviour, observed when Notch signalling is active (S7B Fig.). The amplitude of Her1 protein levels also decreases in the absence of Notch signalling as a consequence of a reduction in gene expression. This drop in gene expression is around 20% (data not shown) in agreement with [[48](#_ENREF_48)] and suggests that the coupling effect that Notch provides is of the right order of magnitude.
