# Supplementary figures and images for: Stochastic Regulation of her1/7 Gene Expression Is the Source of Noise in the Zebrafish Somite Clock Counteracted by Notch Signalling
Source: PLoS Comput Biol. 2015 Nov 20;11(11):e1004459. doi: 10.1371/journal.pcbi.1004459 (PMC4654481; doi:10.1371/journal.pcbi.1004459)

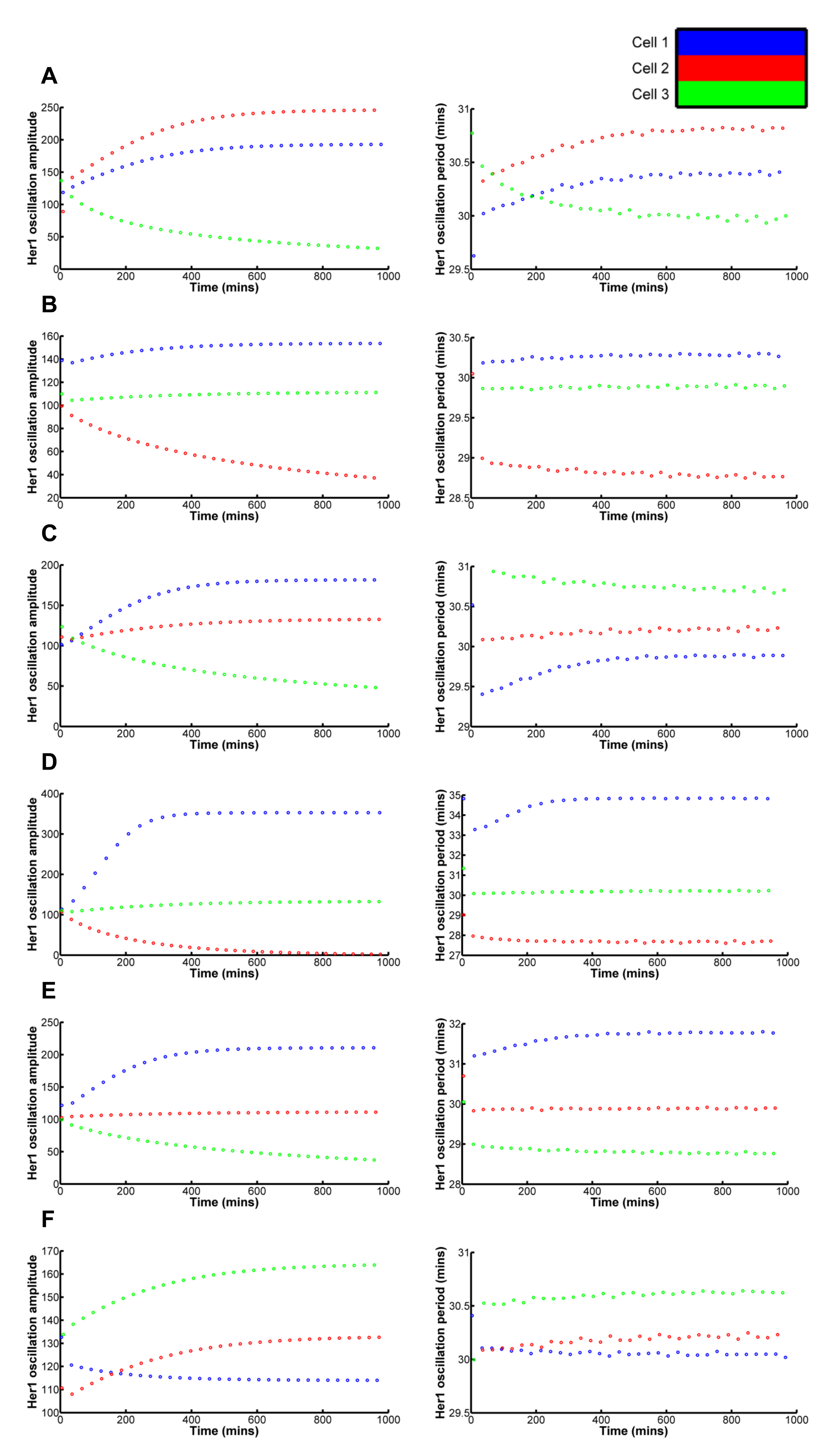

Supplement: S1 Fig — Scatter plots of Her1 oscillation amplitude and period versus time, with colour denoting three different cells selected from Fig 1, to demonstrate variability. S1A: Variability in transcription rate. S1B: Variability in translation rate. S1C: Variability in degradation rate. S1D: Variability in transcription delay. This has the largest effect on period of oscillation. S1E: Variability in translation delay. S1F: Variability in cellular numbers of Hes6 molecules. The plots demonstrate that the amplitude and period of each cell’s oscillation smoothly tend to constants, dependent on the magnitude of the rate/delay/Hes6 constant in that single cell. Once the amplitude/period has reached these constants, independent of other cells, the amplitude and period of that cell will remain fixed for all time. Inter-cellular variability in transcription rate and delay dominate variability in amplitude; inter-cellular variability in transcription delay dominates variability in period. (TIF) [file pcbi.1004459.s005.tif]

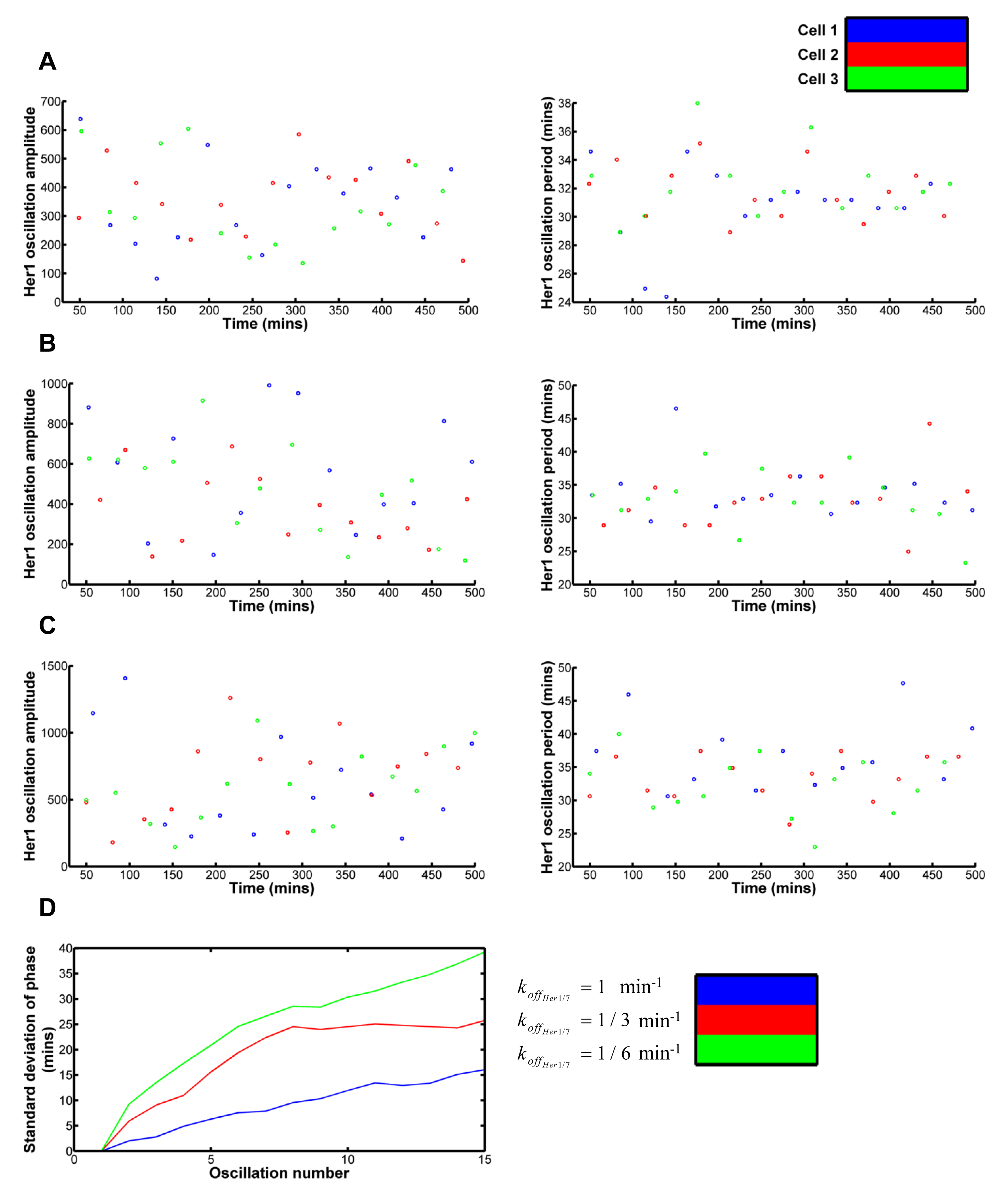

Supplement: S2 Fig — Scatter plots of Her1 oscillation amplitude and period versus time, with colour denoting three different cells randomly selected from Fig 2. S2A: koffHer1/7=1 min-1. S2B: koffHer1/7=1/3 min-1. S2C: koffHer1/7=1/6 min-1. In all three cases the mean amplitude and period for each cell remain fairly constant over time. However, there exist random fluctuations, independent of oscillation stage, around these mean levels. S2D: Plot of standard deviation (20 cells) of phase versus oscillation number for koffHer1/7=1 min-1 (blue), koffHer1/7=1/3 min-1 (red) and koffHer1/7=1/6 min-1 (green). Variability in phase builds up over time. This is slowest for koffHer1/7=1 min-1 and fastest for koffHer1/7=1/6 min-1. Random fluctuations in period of oscillation build up over time, increasing variation in phase of oscillation, causing neighbouring cells to desynchronise. This increase in variation is more rapid the greater the level of stochasticity in gene regulation. (TIF) [file pcbi.1004459.s006.tif]

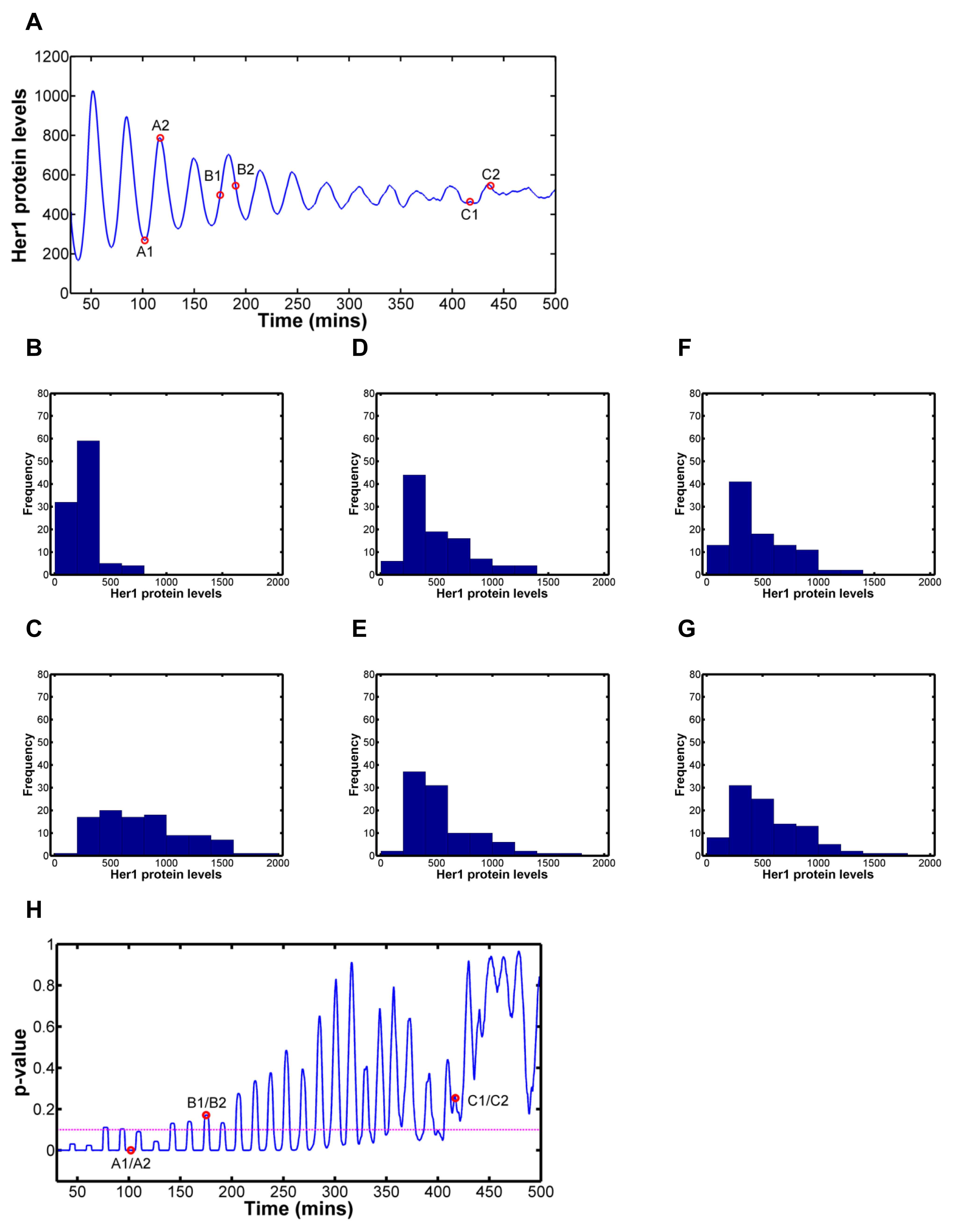

Supplement: S3 Fig — S3A: Mean Her1 protein levels for 100 cells with koffHer1/7=1/2 min-1 (and blocked Notch signalling) as in Fig 2C. S3B-S3G: Histograms of Her1 protein levels at time points indicated by A1, A2, B1, B2, C1, C2 respectively. When in synchrony, the distribution at low Her1 protein levels (S3B) is noticeably different from the distribution at peak Her1 protein levels (S3C). The difference in distribution when moving from trough to peak Her1 levels (S3D) is not noticeably different to the distribution when moving from peak to trough Her1 protein levels (S3E). As the cells desynchronise, the distributions become indistinguishable (S3F-G). S3H: Plot of p-value versus time for Kolmogorov-Smirnov test comparing distribution of Her1 protein levels to distribution of Her1 protein levels 16 minutes later (approximately half an oscillatory period). For early time, the local minima correspond to p-values comparing trough (e.g. A1) to peak (e.g. A2) and the local maxima to comparing a region between trough and peak (B1) to a region between peak and trough (B2). As the cells desynchronise the p-value becomes large (compare distribution at C1 to at C2). We define the cells to have desynchronised from the first point in time when there is not a significant difference (p-value>0.1) between peak distribution and trough distribution (406 minutes in this case). (TIF) [file pcbi.1004459.s007.tif]

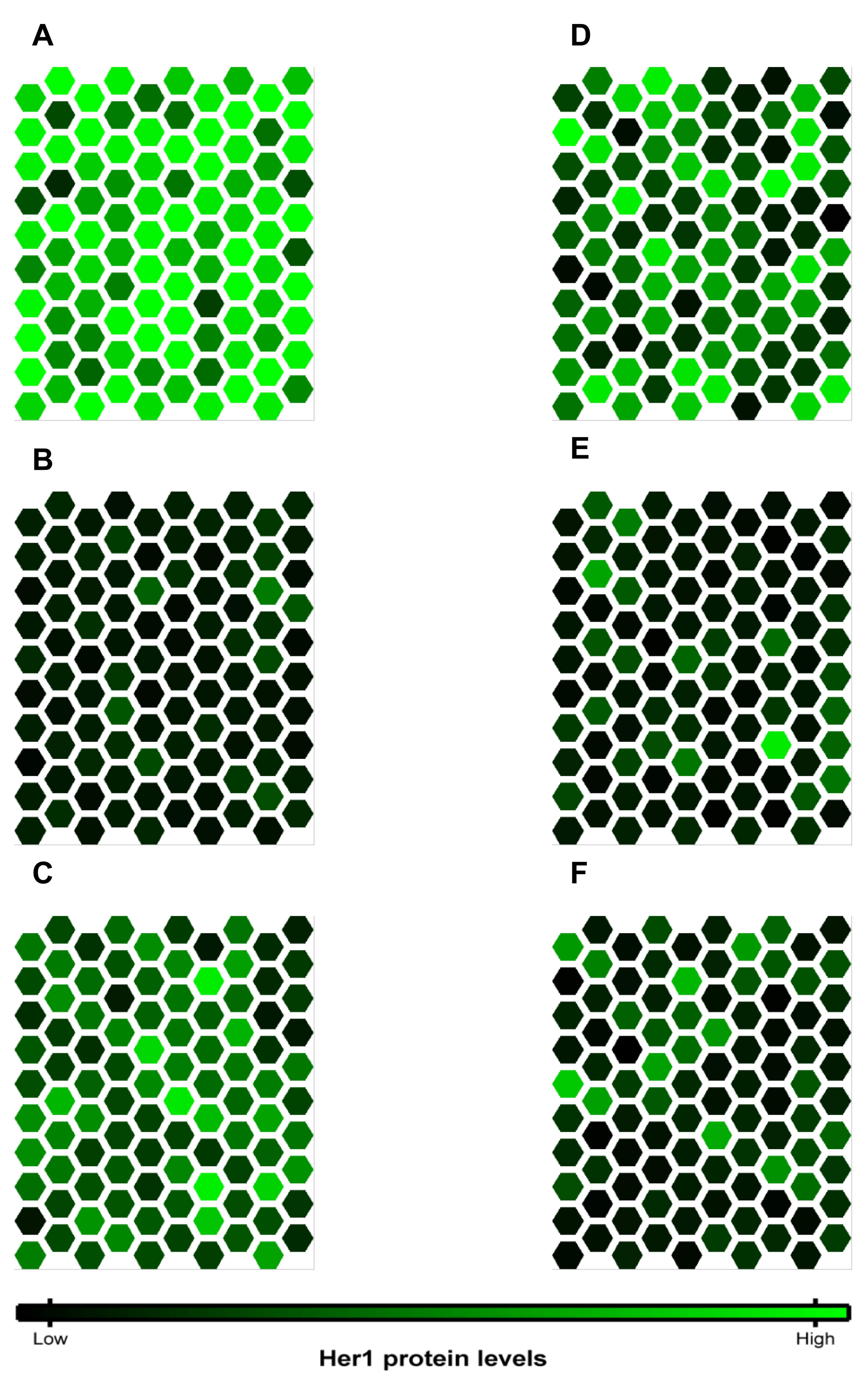

Supplement: S4 Fig — Using data of Fig 2, we compare low levels of stochasticity (A-C) and high levels of stochasticity (D-F) in gene expression (D-F). Initial conditions are identical in all cells. The time points are selected to represent equivalent points in the oscillatory cycle. A (51 mins) and D (53 mins): peaks of the first oscillations. Cells for both koffHer1/7 values are in synchrony. B (132 mins)and E (139 mins): troughs of the third oscillations. koffHer1/7=1/6 min-1 is desynchronising faster than koffHer1/7=1 min-1. C (212 mins) and F (220 mins): peak of the sixth oscillations. The cells for koffHer1/7=1 min-1 are still in synchrony whilst the cells for koffHer1/7=1/6 min-1 demonstrate a salt and pepper pattern. The cell clocks drift out of synchrony very slowly for koffHer1/7=1 min-1, the cells do not desynchronise over this time interval and the salt and pepper pattern of her1 expression is not reached. The cell clocks drift out of synchrony very quickly for koffHer1/7=1/6 min-1, the cells have desynchronised well before the sixth oscillation and a salt and pepper pattern is apparent. (Compare to the wildtype case of S6A–S6C Fig). (TIF) [file pcbi.1004459.s008.tif]

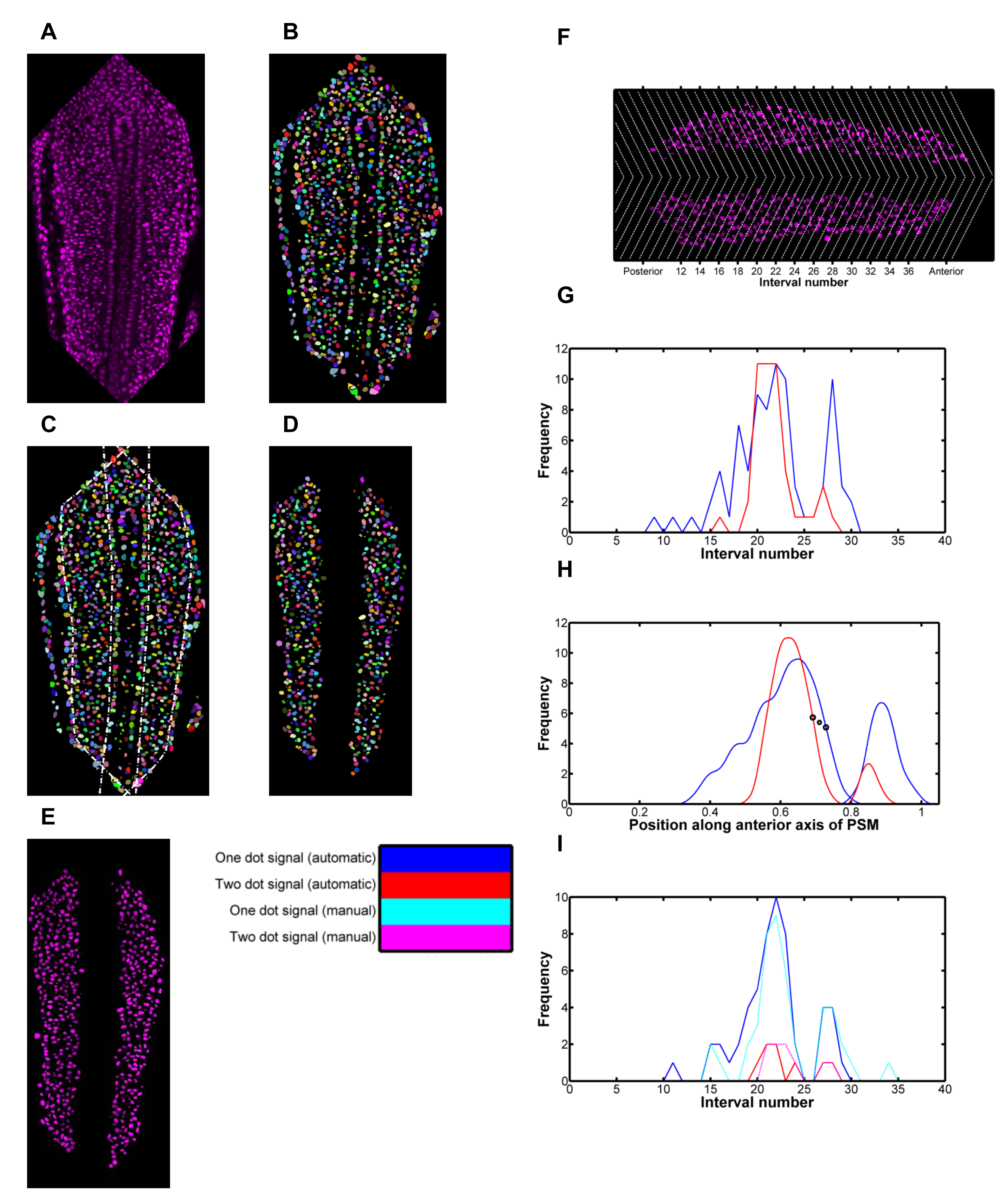

Supplement: S5 Fig — S5A: Single slice of 3D stack of embryo. S5B: The results of segmentation of nuclei for the 3D stack shown for this single slice. Each segmented nuclei is shown in a random colour. S5C: The notochord and embryo boundaries are manually determined for a number of slices in the 3D stack and the results interpolated throughout. S5D: The resulting slice with nuclei within the notochord, out of the embryo boundary or with too much of the nuclei touching the stack boundary removed. The remaining nuclei are those that we analyse. S5E: The slice with only the nuclei that we analyse, and only her1 mRNA transcripts that fall within these nuclei included in the image. S5F: The stack is divided into 40 intervals from the posterior of the PSM to the anterior. The gradient of the intervals are based on the gradient of the her1 mRNA waves. The frequency of nuclei with one dot and two dots is quantified for each interval. S5G: The resulting quantification of frequency of nuclei with one dot and two dots, per interval. S5H: The smoothed signals of S5G Fig. The two dot signal is clearly delayed behind the one dot signal. S5I: Comparison of automatic quantification to manual quantification for the same embryo. A manual count of the number of dots per nuclei in each interval for the innermost single slice of the embryo versus the count per interval in this slice, derived from the three dimensional quantification. The signals are similar, suggesting that the automatic quantification is accurate. (TIF) [file pcbi.1004459.s009.tif]

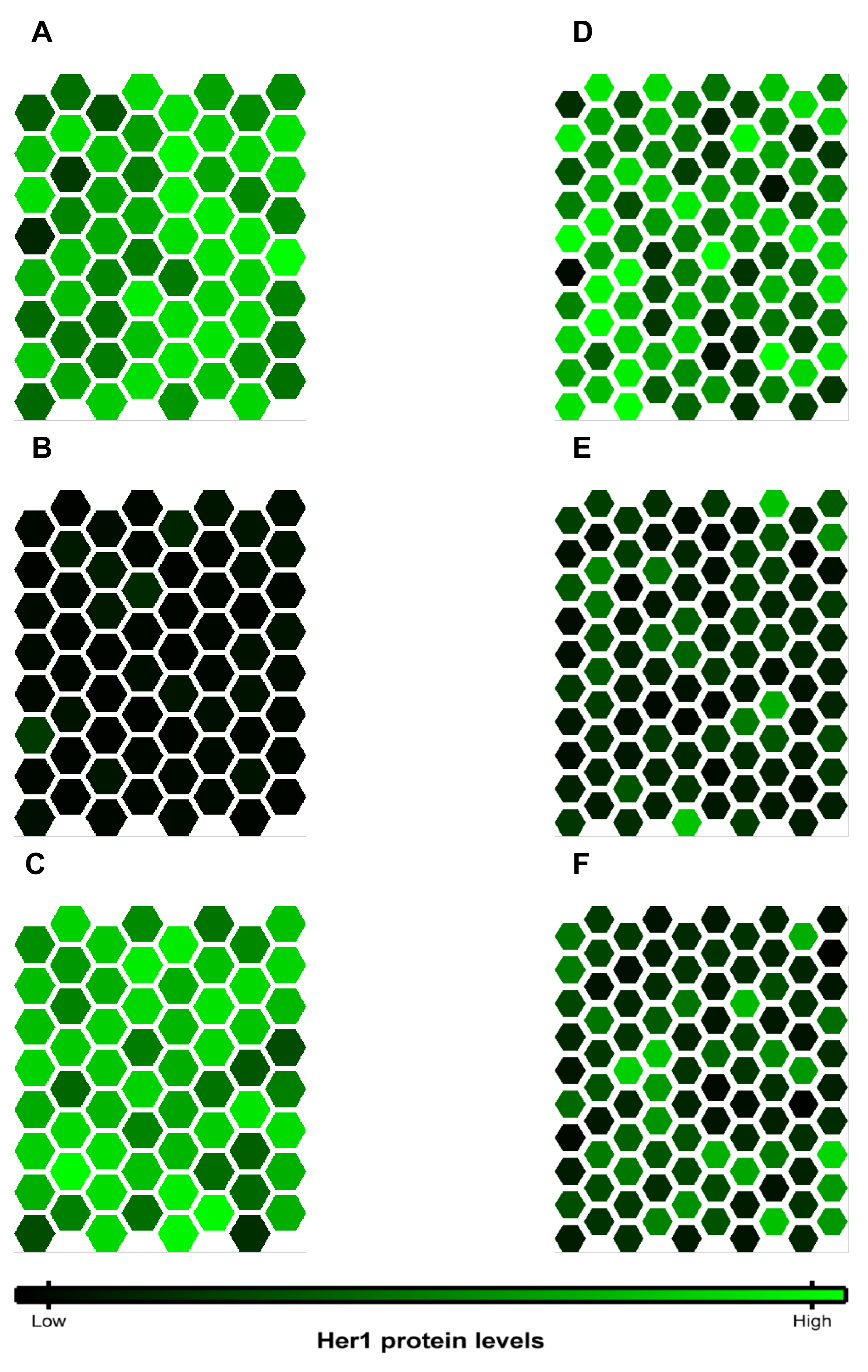

Supplement: S6 Fig — Data is from Figs 2 and 5. Activated Notch signalling (A-C) is compared to the case of deactivated Notch signalling (D-F). The dissociation rate is set as koffHer1/7=1/3 min-1. A (216 mins) and D (52 mins): peaks of oscillation one. In the case of active Notch signalling, oscillation one has been arbitrarily defined as an oscillation after Notch signalling has overridden random initial conditions. The cells oscillate in synchrony in both cases. B (388 mins) and E (200 mins): troughs of oscillation 5. The cells for active Notch oscillate in synchrony whilst the cells for deactivated Notch are drifting out of synchrony. C (518 mins) and F (325 mins): The cells for active Notch oscillate in synchrony whilst those for deactivated Notch have desynchronised and a salt and pepper pattern is apparent. Notch signalling keeps neighbouring cells oscillating in synchrony whilst, in its absence a salt and pepper pattern gradually emerges. (TIF) [file pcbi.1004459.s010.tif]

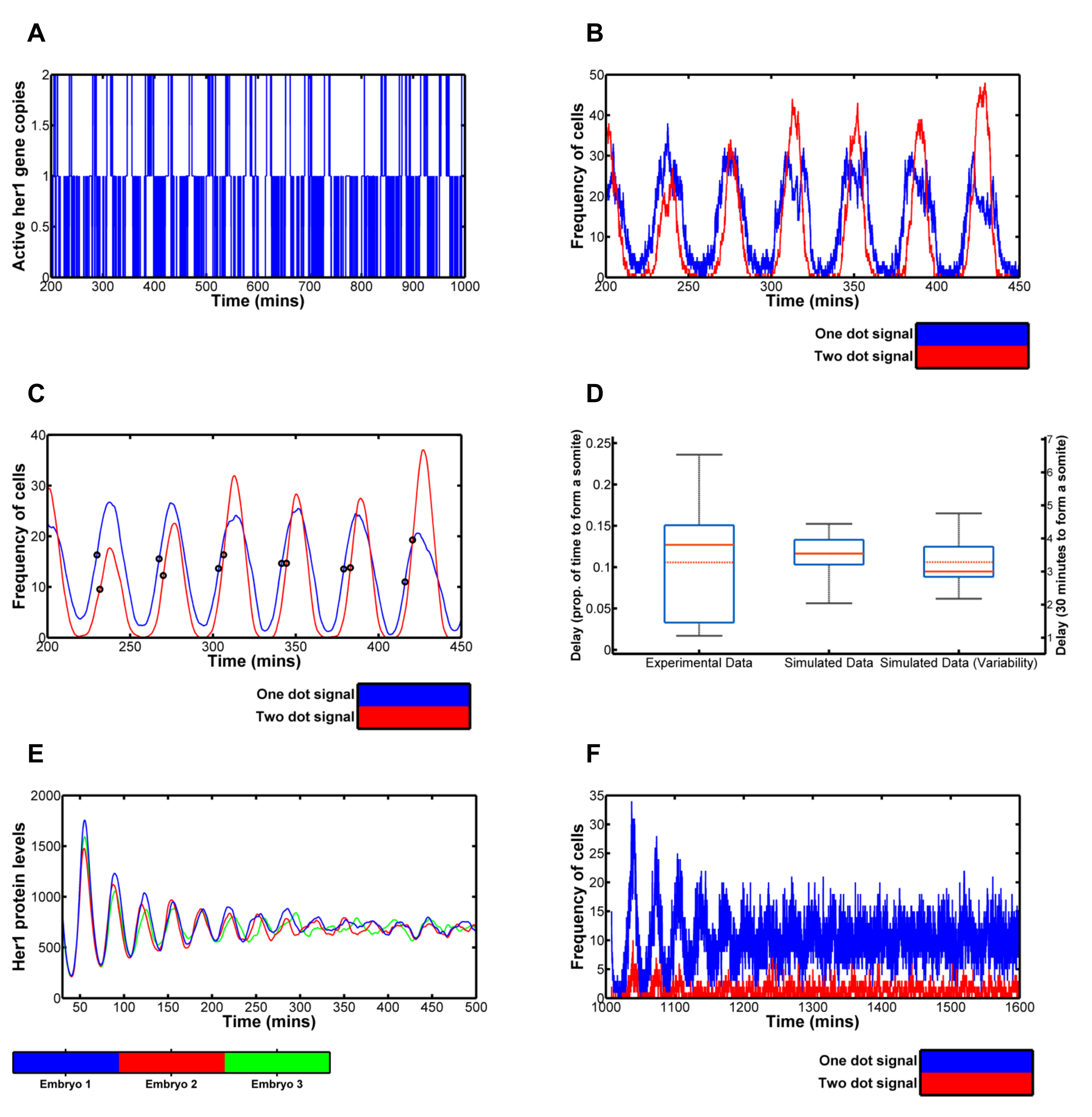

Supplement: S7 Fig — S7A–S7D Fig correspond to active Notch signalling. S7A: Number of active her1 genes versus time, for a single cell. S7B: Plot of frequency of cells with one her1 gene copy expressed (blue signal) and two her1 gene copies expressed (red signal) for the output of the mathematical model (as was done for the experimental data). S7C: Smoothed version of S7B. S7B and S7C Fig demonstrate that there is a delay between the expression of the first her1 gene copy and the second in a cell. The black circles in S7C Fig demonstrate the inflection points used to calculate the delay. S7D: Box and whisker plots comparing sample delays of experimental data (left) to simulated data with stochastic gene regulation (centre) and simulated data with stochastic gene regulation and inter-cellular variability (right). The inter-cellular variability is introduced in the transcription, translation and degradation rates and number of Hes6 molecules at the magnitudes described in Fig 1. The whiskers give the maximum and minimum, the box the lower quartile and upper quartile, the solid red line the median and the red dot-dash line the mean. The left axis gives the scale in terms of proportion of time to form one somite, the right in terms of minutes, on the assumption it takes 30 minutes to form one somite. The boxplots demonstrate that the average delay is well recreated by the mathematical model incorporating stochastic gene regulation, with or in the absence of inter-cellular variability, but the probability distribution is not. The lack of impact when adding inter-cellular variability suggests that delay in gene expression is a consequence of stochasticity in repressor/DNA dissociation and that the quantification of the stochastic dissociation rate is robust to inter-cellular variability. S7E: Plots of mean Her1 protein levels for 100 cells for three different embryos when Notch signalling is deactivated for the case of stochastic gene regulation and inter-cellular variability as in S7D Fig. [file pcbi.1004459.s011.tif]
