# Supplementary material for: Stochastic Regulation of her1/7 Gene Expression Is the Source of Noise in the Zebrafish Somite Clock Counteracted by Notch Signalling
Source: PLoS Comput Biol. 2015 Nov 20;11(11):e1004459. doi: 10.1371/journal.pcbi.1004459 (PMC4654481; doi:10.1371/journal.pcbi.1004459)
Supplement: S1 Table — Expressed in terms of proportion of time to form one somite. (DOCX) [file pcbi.1004459.s012.docx]

**S1 Table. Statistics of the samples of delay from both the experimental data and the simulated data with and without inter-cellular variability**.

|  | Experimental Data | Simulated data | Simulated data with parameter variability |
| --- | --- | --- | --- |
| Mean | 0.106 | 0.116 | 0.106 |
| Median | 0.127 | 0.116 | 0.095 |
| Standard deviation | 0.070 | 0.023 | 0.030 |
| Maximum | 0.236 | 0.152 | 0.165 |
| Minimum | 0.017 | 0.056 | 0.062 |
