# Supplementary material for: Stochastic Regulation of her1/7 Gene Expression Is the Source of Noise in the Zebrafish Somite Clock Counteracted by Notch Signalling
Source: PLoS Comput Biol. 2015 Nov 20;11(11):e1004459. doi: 10.1371/journal.pcbi.1004459 (PMC4654481; doi:10.1371/journal.pcbi.1004459)
Supplement: S2 Table — Expressed in terms of proportion of time to form one somite. (DOCX) [file pcbi.1004459.s013.docx]

**S2 Table. Raw delay data for both the experimental data and the simulated data with and without inter-cellular variability.**

| Experimental Data | Simulated Data | Simulated data with parameter variability |
| --- | --- | --- |
| 0.017 | 0.0562 | 0.0617 |
| 0.020 | 0.0851 | 0.0748 |
| 0.026 | 0.0948 | 0.0844 |
| 0.035 | 0.0959 | 0.0882 |
| 0.044 | 0.0980 | 0.0885 |
| 0.127 | 0.1048 | 0.0893 |
| 0.127 | 0.1107 | 0.0913 |
| 0.132 | 0.1114 | 0.0980 |
| 0.133 | 0.1118 | 0.1147 |
| 0.148 | 0.1125 | 0.1222 |
| 0.159 | 0.1164 | 0.1248 |
| 0.172 | 0.1170 | 0.1248 |
| 0.236 | 0.1200 | 0.1581 |
|  | 0.1285 | 0.1650 |
|  | 0.1315 |  |
|  | 0.1323 |  |
|  | 0.1354 |  |
|  | 0.1356 |  |
|  | 0.1404 |  |
|  | 0.1481 |  |
|  | 0.1524 |  |
